# An in-cluster Sfp-type phosphopantetheinyl transferase instead of the *holo*-ACP synthase activates the granaticin biosynthesis under natural physiological conditions

**DOI:** 10.3389/fchem.2022.1112362

**Published:** 2022-12-22

**Authors:** Ming-Rong Deng, Sin Yu Chik, Yan Li, Honghui Zhu

**Affiliations:** Key Laboratory of Agricultural Microbiomics and Precision Application (MARA), Guangdong Provincial Key Laboratory of Microbial Culture Collection and Application, Key Laboratory of Agricultural Microbiome (MARA), State Key Laboratory of Applied Microbiology Southern China, Institute of Microbiology, Guangdong Academy of Sciences, Guangzhou, China

**Keywords:** phosphopantetheinyl transferase, granaticin, type II polyketide synthase, *gra-orf32*, acyl carrier protein, crosstalk, *Streptomyces vietnamensis*

## Abstract

Bacterial aromatic polyketides are mainly biosynthesized by type II polyketide synthases (PKSs). The PKSs cannot be functional unless their acyl carrier proteins (ACPs) are phosphopantetheinylated by phosphopantetheinyl transferases (PPTases). Gra-ORF32 was identified as an in-cluster PPTase dedicated for granaticin biosynthesis in *Streptomyces vietnamensis* and the Arg- and Pro-rich N terminus was found to be crucial for catalytic activity. Overexpression of the encoding genes of the *holo*-ACP synthases of fatty acid synthases (FAS ACPSs) of both *E. coli* and *S. vietnamensis* could efficiently activate the production of granaticins in the Δ*gra-orf32* mutant, suggesting the ACP of granaticin (graACP) is an efficient substrate for FAS ACPSs. However, Gra-ORF32, the cognate PPTase of the graACP, could not compensate the conditional deficiency of ACPS in *E. coli* HT253, indicating that it has evolved to be functionally segregated from fatty acid biosynthesis. Nine out of eleven endogenous and all the tested exogenous non-cognate PPTases could activate the production of granaticins to varied extents when overexpressed in the Δ*gra-orf32* mutant, indicating that ACPs of type II PKSs could also be widely recognized as effective substrates by the Sfp-type PPTases. The exogenous PPTases of type II PKSs activated the production of granaticins with much higher efficiency, suggesting that the phylogenetically distant in-cluster PPTases of type II PKSs could share substrate preferences for the ACPs of type II PKSs. A significantly elevated production of granaticins was observed when the mutant Δ*gra-orf32* was cultivated on ISP2 plates, which was a consequence of crosstalk between the granaticin pathway and a kinamycin-like pathway as revealed by transcriptome analysis and pathway inactivations. Although the host FAS ACPS could efficiently activate the production of granaticins when overexpressed, only Gra-ORF32 activated the efficient production of granaticins under natural physiological conditions, indicating that the activity of the host FAS ACPS was strictly regulated, possibly by binding the FAS *holo*-ACP product with high affinity. Our findings would contribute to a more comprehensive understanding of how the ACPs of type II PKSs are activated and facilitate the future functional reconstitutions of type II PKSs in *E. coli*.

## Introduction

Bacterial aromatic polyketides are a class of natural products with diverse bioactivities and chemical structures, some of which have been developed as front-line antibiotics and anticancer drugs, making them very important in the pharmaceutical industry and the clinics (Katz and Baltz, 2016). This category of natural products is mainly biosynthesized by the type II polyketide synthases (PKSs). A *β*-ketoacyl synthase (KS), a chain length factor and an acyl carrier protein (ACP) constitute the so-called iterative minimal PKS that is responsible for synthesis of linear polyketide chains with varied length (Hopwood, 1997). The ACP is inactive unless it undergoes a post-translational modification process, in which phosphopantetheinyl transferases (PPTases) catalyze the transfer of 4′-phosphopantetheine (PPant) arms from coenzyme A (CoA) to conserved serine residues of *apo*-ACP through phosphoester bonds. The resulting active *holo*-ACPs covalently yet transiently tether the substrates and the growing polyketide chains on the “prosthetic” PPant arms, assisting the KSs as hubs to catalyze the programmed elongations of polyketide chains (Lambalot et al., 1996; Beld et al., 2014).

Based on sequences, structures, and their target-elongating synthases, the PPTase superfamily has been classified into three families: the the *holo*-ACP synthases (ACPSs), the Sfp-type PPTases and the type I integrated PPTases (Lambalot et al., 1996; Beld et al., 2014). The first two families are discrete PPTases, usually activating carrier proteins of type II fatty acid synthases (FASs) and biosynthetic synthases of secondary metabolism, respectively. The third family of PPTases are translationally incorporated as a catalytic domain at the C terminus of type I FASs or some biosynthetic megasynthases of secondary metabolism (Beld et al., 2014). In contrast to extensive studies on the PPTases involved in activation of the carrier proteins of type I PKS and non-ribosomal peptide synthetase (NRPS) pathways (Quadri et al., 1998; Sánchez et al., 2001; Volokhan et al., 2005; Jiang et al., 2013; Tufar et al., 2014; Wang et al., 2016; Kim et al., 2018), PPTases that activate ACPs of type II PKS pathways have been less investigated. Early studies showed that the *Escherichia coli* ACPS could install PPant arms to *apo*-ACPs from several type II polyketide pathways *in vitro*, including the ACP of granaticin (graACP), and these enzymatically synthesized *holo*-ACPs could functionally work with KS to produce the same aromatic polyketides as those from the natural minimal PKS *in vivo* (Carreras et al., 1997; Gehring et al., 1997). Co-expression of the ACPS gene of the fatty acid synthase (FAS ACPS) of *E. coli* with the ACP genes of actinorhodin and griseusin in *E. coli* leading to high levels of production of active *holo*-ACPs was also reported (Cox et al., 1997), suggesting that FAS ACPSs possess remarkable substrate promiscuity towards ACPs of type II PKS pathways. Another fact is that the model type II polyketide cluster of actinorhodin and many other clusters of aromatic polyketides lack apparent cluster-situated PPTase genes. Thus, the perspective that ACPs of type II PKSs are usually activated by the hosts’ own FAS ACPSs has been widely acknowledged (Kim et al., 2018).

**FIGURE 1:**
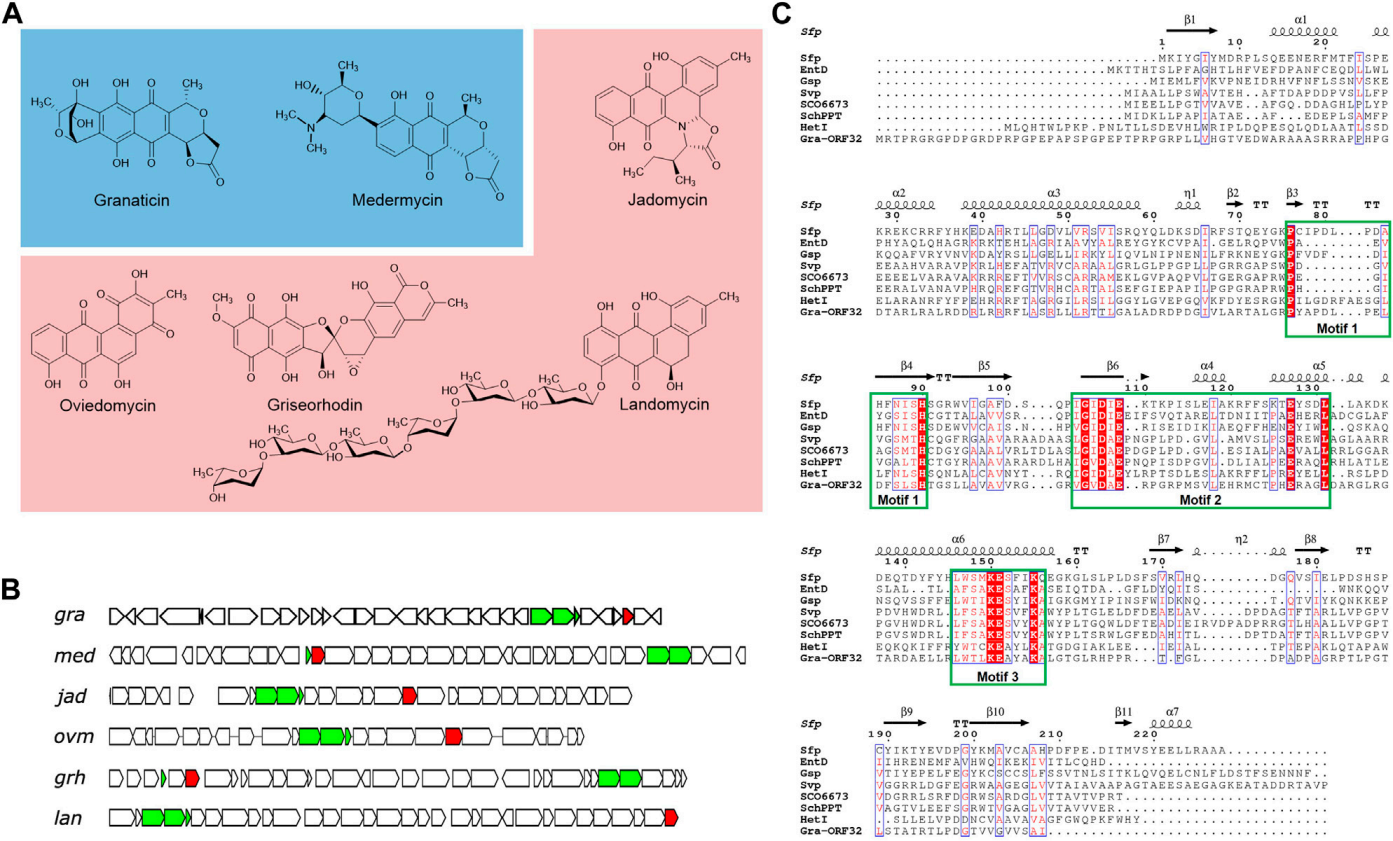
Selected type II polyketides and their biosynthetic gene clusters harboring an in-cluster phosphopantetheinyl transferase (PPTase) gene. **(A)** Representative type II polyketides. Pyranonaphthoquinone members are colored in blue background and angular polyketides in pink. **(B)** Organizations of the biosynthetic gene clusters of the selected type II polyketides, highlighting the minimal PKS and the PPTase genes. The minimal PKS genes are colored in green and the PPTase genes in red. **(C)** Sequence alignment of Gra-ORF32 with Sfp and other Sfp-type PPTases. The surfactin PPTase Sfp is from *Bacillus subtilis* (CAA44858.1) and its crystal structure (PDB: 1QR0) was used to generate the ESPript output picture. Other PPTases, *E. coli* EntD (NP_415115.2), *Brevibacillus brevis* Gsp (CAA53988.1), *S. verticillus* ATCC15003 Svp (AAG43513.1), *S. coelicolor* A3 (2) SCO6673 (WP_011031096.1), *S. chattanoogensis* L10 SchPPT (AFF18625.1) and *Nostoc* sp. PCC 7120 HetI (AAA22003), were included in this alignment. The three conserved motifs, P(x)n (S/T)H(S/C), (V/I)G (V/I)D (L/I/V)E(x)nE and (S/T/C)xKE (S/A)hhK (A/Q) are shown in the square green box.

However, a number of identified type II PKS gene clusters have been found to contain a putative Sfp-type PPTase gene, such as granaticin, medermycin, jadomycin, oviedomycin, griseorhodin and landomycin (Figures 1A,B) (Ichinose et al., 1998; Westrich et al., 1999; Wang et al., 2001; Li and Piel, 2002; Ichinose et al., 2003; Lombó et al., 2004). ACPS-type PPTase genes were also found in some type II PKS gene clusters, with FdmW being the first characterized in-cluster ACPS-type PPTase in the fredericamycin biosynthesis (Huang et al., 2006). Inactivation of the PPTase genes *jadM* and *fdmW* led to major decreases (over 93%) in jadomycin and fredericamycin productions, respectively, suggesting that ACPs from these pathways cannot be activated efficiently *in vivo* by the hosts’ FAS ACPSs (Wang et al., 2001; Huang et al., 2006). Moreover, the ACP of xanthomonadin which is biosynthesized by a type II PKS (Cao et al., 2018) was very recently found to be activated by a Sfp-type PPTase whose encoding gene stands outside of the biosynthetic cluster in *Xanthomonas campestris* pv. *campestris* (Chen et al., 2022). These findings blurred our understanding of how ACPs of type II PKSs are activated. Therefore, more investigations are required for a more comprehensive and in-depth understanding on the activation mechanisms of ACPs of type II PKS pathways.

The biosynthetic gene cluster of granaticin (*gra*) was first identified more than 2 decades ago, and the *gra-orf32* gene was initially annotated with unknown function (Ichinose et al., 1998). The second *gra* cluster was reported by our lab in *Streptomyces vietnamensis* GIMV4.0001 (Deng et al., 2011a). With genomic sequencing of GIMV4.0001, this *gra-orf32* gene was recognized as a putative PPTase-encoding gene (Deng et al., 2015). The facts that the graACP is efficiently activated by *E. coli* FAS ACPS *in vitro* (Gehring et al., 1997), and the *gra* cluster harbors a putative PPTase gene prompted us to carry out a more detailed investigation on it. The results present here might be helpful for better understanding the activation of ACPs of type II PKS pathways.

## Materials and methods

### Strains, plasmids, biochemicals and general procedures

Strains and plasmids constructed by or used in this study were listed in Supplementary Table S1. Polymerase chain reaction (PCR) primers were ordered from and synthesized by Azenta China, and listed in Supplementary Table S2. *E. coli* NEB Turbo and ET12567/pUZ8002 (MacNeil et al., 1992) were used for general cloning and intergeneric conjugation, respectively. *E. coli* HT253 (Takiff et al., 1992) was used to evaluate possible functional complementation of the *E. coli* FAS ACPS. *S. vietnamensis* GIMV4.0001 (Zhu et al., 2007) is a wild-type granaticin producer. The plasmid pKC1139 (Bierman et al., 1992) was used for in-frame deletion of *gra-orf32*. Other disruption mutants were made based on the pYH7 plasmid instead (Sun et al., 2006). An integrative plasmid pSETAT-KasOp*, a gift from Prof. Keqiang Fan, was used for the gene complementation and overexpression purposes. pSETAT-KasOp* is derived from pSET-KasOp* (Pan et al., 2017) with ampicillin and thiostrepton resistances in *E*. *coli* and *Streptomyces*, respectively. Phanta Max Super-Fidelity DNA polymerase, ClonExpress MultiS One Step Cloning Kit, gel extraction and plasmid preparation Kits were products of Vazyme Biotech Co., Ltd., China. The experiments were performed according to the manufacturer’s procedures. Other common biochemicals and medium components were purchased from routine commercial sources. Gene synthesis and DNA sequencing were performed by Azenta China. *E. coli* strains containing plasmids were cultured in LB medium at 37°C with 200 rpm agitation, supplemented with appropriate antibiotics as required. *Streptomyces* strains were grown at 30 °C on ISP2 agar medium for sporulation or in liquid TSB for growth of mycelium. The liquid YEME medium (0.3% yeast extract, 0.5% tryptone, 0.3% malt extract, 1% glucose, 5 mM MgCl_2_) was used for isolation of genomic DNA and total RNA and granaticin production. Gauze’s synthetic medium No.1, ISP2 and MS were also used for parallel analysis or other purposes when necessarily. *E. coli*-*Streptomyces* conjugations were performed on either ISP4 or MS agar plates.

### Bioinformatic analyses

The sequence alignment of Sfp-type PPTases was performed by using the online MultAlin program (Corpet, 1988), and the final output was processed by ESPript 3.0 (Robert and Gouet, 2014) using the crystal structure of Sfp (PDB: 1QR0) (Reuter et al., 1999) for secondary structure depiction. Minimum evolution phylogenetic tree was generated by the Mega11 program (Tamura et al., 2021). The EFI - ENZYME SIMILARITY TOOL (EFI-EST) and the EFI - GENOME NEIGHBORHOOD TOOL (EFI-GNT) (Zallot et al., 2019) were used for search of type II PKSs with Sfp-type PPTase genes. To do this, the sequence of Gra-orf32 was used as the query sequence to BLAST the Uniprot database with the EFI-EST. The BLAST query e-value and the maximum number of sequences retrieved were set as default. The retrieved sequence similarity network (SSN) file was then submitted to the EFI-GNT. The neighbourhood size was set as 15.

### In-frame deletion in *S. vietnamensis*

According to the organization of the *gra* cluster (Deng et al., 2011a), the putative PPTase gene *gra-orf32* and the cyclase gene *gra-orf33* are co-transcribed since the non-coding sequence between these two genes is too short to harbour a typical promoter. To avoid the potential polar effects on downstream genes, an in-frame deletion of *gra-orf32* was desired. Two 1.8-kb DNA fragments flanking the *gra-orf32* gene were amplified and ligated into the linear vector pKC1139 digested with *Hin*dIII and *Eco*RI, affording the disruption plasmid pKC-Δgra-orf32. All the constructed plasmids are confirmed by DNA sequencing to ensure that no mutation was introduced during the manipulation process. The disruption plasmid pKC-Δgra-orf32 was then introduced into the non-methylating *E. coli* ET12567/pUZ8002, conjugated to *S*. *vietnamensis* GIMV4.0001 following a previous protocol (Deng et al., 2011b). Exconjugants were picked and re-streaked on nutrient agar plates supplemented with 30 μg/ml apramycin and 25 μg/ml nalidixic acid and grown at 30 °C for 1 day. Single colonies were then inoculated into liquid YEME medium. After several rounds of liquid cultivation without antibiotics at 37°C, the temperature-sensitive plasmid pKC-Δgra-orf32 are expected to be lost. The cultures were then diluted and spread on nutrient agar plate. The colonies that are sensitive to apramycin were picked and subjected to DNA isolation and PCR verification. The mutants Δ*gra-orf32*Δ*kina-like-pks*, Δ*gra-orf32*Δ*pig-pks* and Δ*gra-orf32*Δ*kina-like-pks*Δ*pig-pks* were generated using pYH7-based recombinant plasmids in a similar way, except for the relaxed cultivation of exconjugants to lose the disruption plasmids where the cultivation temperature is 30 °C as normal.

### Complementation and introducing other genes into *S. vietnamensis*

For complementation or introducing other genes into *S. vietnamensis*, the target genes were amplified and then assembled individually, using the ClonExpress MultiS One Step Cloning Kit, into pSETAT-KasOp* between the *Afl*II and *Spe*I sites where the open reading frame (ORF) was immediately downstram the promotor KasOp*. The resulting plasmids were then individually introduced into the Δ*gra-orf32* mutant or other strains with the same method as described in the in-frame deletion section. Screening of the desired colonies followed the standard procedure. The *E. coli* FAS ACPS gene and the PPTase gene *sfp* of surfactin biosynthesis of *Bacillus subtilis* were codon-optimized and synthesized by Azenta China (Supplementary Figures S1,2). The PPTase genes *jadM* and *med-orf24* of jadomycin and medermycin biosynthetic pathways were also synthesized since these biological materials are not available in our lab. The synthesized genes were cloned into pSETAT-KasOp* between the *Afl*II and *Spe*I sites as same as other genes.

### Expression of *gra-orf32 in E. coli* HT253

The pET28b (+) plasmid was used for heterologous expression in *E. coli*. The *gra-orf32* gene was codon-optimized (Supplementary Figure S3), synthesized and then cloned into pET28b (+) between the *Nde*I and *Xho*I sites, affording the expression plasmid pET28b-gra-orf32. The plasmid pET28b-gra-orf32 was transformed into *E. coli* HT253. HT253 is a mutant with a mini-Tn10 insertion in the *pdxJ* gene which is immediately upstream of and co-transcribed with the FAS ACPS gene *acpS* (Takiff et al., 1992). The growth of HT253 is tetracycline-dependent since the original transcript was interrupted and two divergent tetracycline-inducible promoters were introduced by the mini Tn10 insertion. The putative FAS ACPS gene (*SVTN_RS23020*) in *S. vietnamensis* was also codon-optimized (Supplementary Figure S4), synthesized and cloned into pET28b (+) in the same way for comparison. Further analysis found another ACPS-type gene (*SVTN_RS21115*) in its genome (Supplementary Table S3), and this gene was also included in the evaluation of possible functional compensation in *E. coli* (Supplementary Figure S5). The growth abilities of HT253 carrying different expression plasmids were tested on LB plate with 50 μg/ml kanamycin and 0.5 mM isopropyl *β*-d-1-thiogalactopyranoside (IPTG) and with/without 5 μg/ml tetracycline. The seed cells prepared from permissive medium were twice washed with sterile water before inoculation.

### Transcriptome analysis

Transcriptome analysis was performed by Novogene through strand-specific RNA sequencing. Each *Streptomyces* cell sample was collected from three plates of either YEME or ISP2 cultures after 24 h cultivation. Total RNA was isolated and used as input material for the RNA sample preparations, then the mRNA samples were obtained by using probes to remove rRNA. Strand-specific RNA-seq libraries were generated using NEBNext^®^ UltraTM RNA Library Prep Kit for Illumina^®^ (NEB, United States) following manufacturer’s recommendations. The quality of library was assessed on the Agilent Bioanalyzer 2,100 system. The sequencing was performed on an Illumina Novaseq platform. HTSeq v0.6.1 was used to count the reads numbers mapped to each gene. And then FPKM of each gene was calculated based on the length of the gene and reads count mapped to this gene. Differential expression was performed using the DESeq R package (1.18.0). The resulting *p*-values were adjusted using the Benjamini and Hochberg’s approach for controlling the false discovery rate. Genes with an adjusted *p*-value < 0.05 found by DESeq were assigned as differentially expressed. All samples were analysed in triplicate.

### Chemical analysis and quantification of granaticin production

The methods for sample preparation and analysis were similar to those of our previous works (Deng et al., 2020; Deng et al., 2021), but analyses were conducted on different apparatuses. For fermentation cultures, 5% (w/v) resins (Amberlite^®^ XAD16, Shanghai Macklin Biochemical Co., Ltd., China) were added into the cultures at the end of fermentation. After a 3-h extended incubation with shaking to allow metabolites to be absorbed into resins, then the resins were harvested, cleaned and air-dried. Metabolites were extracted with methanol from resins. The extracts were evaporated and re-dissolved in methanol. For plate cultures, the medium agar and the cells were together chopped and extracted with ethyl acetate, the extracts were also evaporated and re-dissolved in methanol. HPLC analyses were conducted with a Poroshell HPH-C18 column (4.6 × 250 mm, 4 μm, Agilent) on a Shimadzu LC-20AT system under a gradient elution at a flow rate of 0.6 ml/min. The detailed gradient program was set as follows: 0–10 min, 20% – 40% B; 10–20 min, 40%–50% B; 20–30 min, 50%–100% B; 30–35 min, 100% B, where A is H_2_O with 0.1% formic acid and B is acetonitrile unless otherwise noted. The UV absorbance data were collected at the wavelength of 520 nm. The identities of granaticins were confirmed by both using pure compounds as standards and MS analysis. LC-MS analysis was carried out, with an ESI source in negative ion mode, on an Agilent 6,430 Triple Quad mass spectrometry coupled to 1,290 Infinity LC System equipped with an Agilent Poroshell 120 PheHex column (2.1 × 100 mm, 2.7 μm). Liquid chromatography for LC-MS analysis was performed using a 25 min solvent gradient (0.25 ml/min) from 10% – 100% CH_3_OH in H_2_O containing 0.1% formic acid. For quantification, yield of each compound was measured in moles with a standard calibration of pure compound. The quantities of the four main products, granaticin, granaticin B, granaticinic acid and granaticinic acid B, in each strain were calculated together as the overall granaticin production of this strain. All the quantifications were done in triplicate. Analysis of variance was used to test for statistically significant differences between the groupings of samples (*p* < 0.05).

## Results

### Gra-ORF32 is a typical Sfp-type PPTase by bioinformatic analysis

A comparison of Gra-ORF32 with *B. subtilis* Sfp revealed only 16.5% identity and 28.8% overall similarity in sequence. Despite low level of sequence similarity, which is one of the characteristics of the PPTase superfamily, a further bioinformatic analysis showed that Gra-ORF32 contains three conserved motifs: PYAPDLPELDFSLSH, VGVDAE and WTLKEAYAK and the key residues essential for activity and structural stability (Figure 1C). The three conserved motifs: P(x)n (S/T)H, (V/I/L)G (V/I/L)D (L/I/V) (x)nE and (W/F) (S/T/C/A)xKE (S/A)hhK (where h is an amino acid with a hydrophobic chain) have been found across all the characterized Sfp-type PPTases (Asghar et al., 2011; Beld et al., 2014).

It is notable that the open reading frame (ORF) of *gra-orf32* from *S. vietnamensis* GIMV4.0001 was originally predicted to be 657-bp long, but this version of ORF is dysfunctional. An extended 771-bp version was proved to be active (Supplementary Figure S6, see the next section). Therefore, in comparison to Sfp, Gra-ORF32 has about 33 additional amino acids at the N-terminal end. The situation is similar to that of FdmW, a dedicated ACPS-type PPTase for biosynthesis of the aromatic polyketide fredericamycin (Huang et al., 2006). Another noteworthy similar feature to FdmW is that Gra-ORF32 is rich in Arg (38/256) and Pro (33/256), resulting in a very basic protein. The predicated isoelectric point (pI) of Gra-ORF32 is 11.41, in contrast with the low pIs of other Sfp-type PPTases from non-type-II PKS clusters. A search of type II PKS clusters with Sfp-type PPTase genes from the database by using the EFI-GNT (Zallot et al., 2019) revealed that more than 50 uncharacterized type II PKS clusters harbor at least one Sfp-type PPTase gene (Supplementary Figure S7), indicating that many of type II PKS pathways have evolved to accommodate a specific PPTase-encoding gene in their biosynthetic clusters for activation of their carrier proteins.

### *Gra-orf32* is crucial for efficient production of granaticins

**FIGURE 2:**
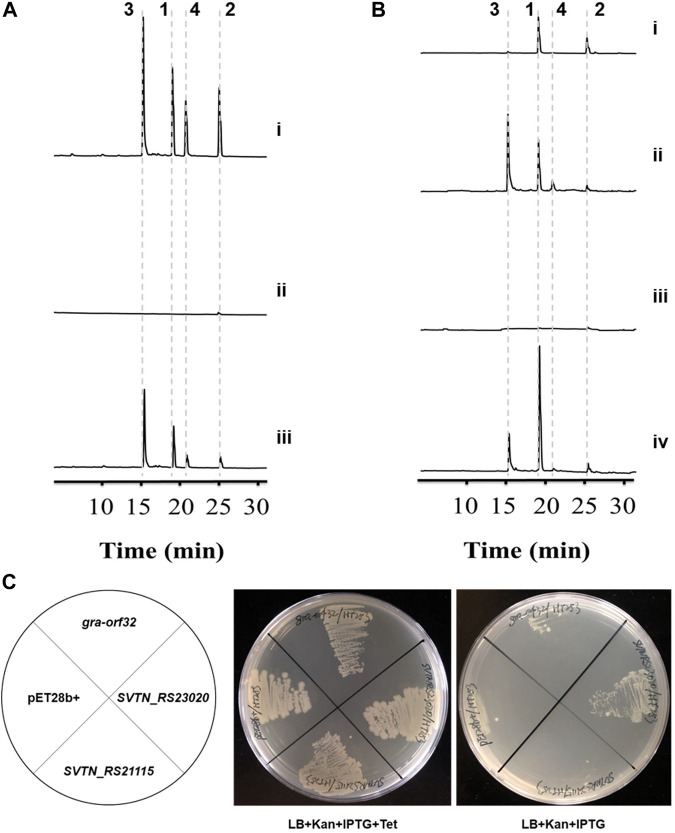
Inactivation, complementation and heterologous expression of the Sfp-type PPTase gene *gra-orf32*. **1**, granaticin; **2**, granaticin B; **3**, granaticinic acid; **4**, granaticinic acid B. The UV absorbance data were collected at the wavelength of 520 nm. **(A)** HPLC metabolic profiling of the in-frame deletion and complementation strains of *S. vietnamensis*. Strains were rotationally cultivated in liquid YEME for 3 d i, the wild-type strain GIMV4.0001; ii, the in-frame deletion mutant Δ*gra-orf32*; iii, the complementation strain Δ*gra-orf32*:*gra-orf32*. **(B)** HPLC metabolic profiling of cultures of the mutant Δ*gra-orf32* from ISP2 and YEME plates. i, the deletion mutant Δ*gra-orf32* on ISP2 plate; ii, the wild-type strain GIMV4.0001 on ISP2 plate; iii, the deletion mutant Δ*gra-orf32* on YEME plate; iv, the wild-type strain GIMV4.0001 on YEME plate. **(C)** Heterologous expression of *gra-orf32* in the conditional ACPS-deficient *E. coli* mutant HT253. The growth of HT253 is tetracycline-dependent. *SVTN_RS23020* is the putative FAS ACPS gene in *S. vietnamensis*. *SVTN_RS21115* is another ACPS-type gene located in a NRPS-like cluster in *S. vietnamensis*. All strains could grow normally on plates with kanamycin, IPTG and tetracycline, but only the strain carrying *SVTN_RS23020* was viable on plates without tetracycline.

The PPTase-encoding gene *gra-orf32* locates in the *gra* cluster, making it reasonable to expect that *gra-orf32* encodes a dedicated PPTase for activation of the graACP. In order to prove it, *gra-orf32* was inactivated with an in-frame deletion method, generating the mutant Δ*gra-orf32*. The genotype of Δ*gra-orf32* was confirmed by PCR verification (Supplementary Figure S8) and DNA sequencing. The wild-type *S. vietnamensis* produces granaticins very well both in liquid and on solid YEME medium. As expected, no obvious blue pigment (the color of granaticins) could be observed with naked eyes from both liquid and solid cultures of Δ*gra-orf32* (Supplementary Figure S9), and only a trace amount of granaticins could be detected by HPLC from liquid YEME cultures of Δ*gra-orf32* (Figure 2A). Then we re-introduced the *gra-orf32* gene into the Δ*gra-orf32* mutant by using a pSET152-based vector with the ORF immediately downstream the strong promoter KasOp* (Supplementary Figure S10). However, introducing the initially predicted ORF of the *gra-orf32* gene could not complement the mutant Δ*gra-orf32*. After a carefully inspection of the sequence, another 114-bp-upstream GTG start codon was found. The complementation strain with this extended ORF of the *gra-orf32* gene successfully restored the production of granaticins (Figure 2A). The production level of granaticins in the complementation strain was about 35%–55% to that of the wild-type strain. However, on plates with YEME and all other tested media, the complementation strain produced apparently more granaticins than the wild-type strain (Supplementary Figure S9). This reverse differences in yields under different cultivation conditions might be due to the differences in the transcriptional activity of the *gra-orf32* gene in wild type strain and its relatively constant transcriptional activity in the complementation strain. The results suggested a crucial role of *gra-orf32* for efficient granaticin production. Thus, Gra-ORF32 is a dedicated PPTase for granaticin biosynthesis.

As mentioned above, the Δ*gra-orf32* mutant still reserved an ability to produce trace amount of granaticins. There were two possibilities accounting for it: i) a latent but low-level activity from other endogenous PPTase(s); ii) crosstalk of granaticin biosynthesis with other type II PKSs. For the second possibility, we indeed obtained some results that could only be attributed to crosstalk between pathways in our previously studies. When we first established the genetic manipulation system on this new granaticin-producing strain, the mutant DMR1 was generated by using the *aac(3)IV* cassette to replace the minimal PKS genes (*gra-orf1*, *gra-orf2*, *gra-orf3*) and no granaticins were detected by HPLC analysis following a routine sample preparation and analysis procedure (Deng et al., 2011b). This DMR1 mutant was later used for discovery of potential new natural products by using an “one strain many compounds” (OSMAC) activation strategy. However, a small amount of granaticin was isolated from a large batch of fermentation of DMR1, which was confirmed by high resolution MS and ^1^H NMR analysis (Lin, 2017). Hence the trace amount production of granaticins by Δ*gra-orf32* might also be a consequence of crosstalk of the granaticin machinery with others.

However, a more complicated phenomenon was observed. When cultivated on ISP2 plates for sporulation, Δ*gra-orf32* produced much more granaticins than in liquid or on solid YEME (Figure 2B). Under this cultivation condition, the expression of some latent PPTase gene(s) might be elevated or another pathway that could crosstalk with the granaticin pathway was activated instead, or both. Thus, the exact underling mechanism remains to be investigated.

### Gra-ORF32 could not functionally compensate the FAS ACPS of *E. coli*

The *E. coli* FAS ACPS was reported to catalyze PPant transfer *in vitro* to a variety of ACPs from *Streptomyces*-origin type II PKS pathways (Gehring et al., 1997). Amongst the tested type-II-PKS ACPs, graACP is the best non-cognate substrate of the *E. coli* FAS ACPS (Gehring et al., 1997). To probe whether the Sfp-type PPTases and the ACPSs that can efficiently phosphopantetheinylate the same ACP of type II PKS have similar substrate specificity for the FAS ACPs, we expressed *gra-orf32* in an *E. coli* mutant HT253. HT253 is a conditional ACPS-deficient mutant (Takiff et al., 1992). It grows normally in LB with tetracycline, but doesn’t grow in LB without tetracycline. The *gra-orf32* gene was codon-optimized and cloned into pET28b (+). The putative FAS ACPS gene (*SVTN_RS23020*) of *S. vietnamensis* and another ACPS-type gene (*SVTN_RS21115*) were also expressed for comparison. All the HT253 strains were viable on plates with kanamycin, IPTG and tetracycline. In contrast, only the strain carrying the plasmid pET28b-SVTN_RS23020 could grow at a lower rate than normal on plates without tetracycline (Figure 2C). The results showed that Gra-ORF32 could not functionally compensate the FAS ACPS in *E. coli*, suggesting that the Sfp-type Gra-ORF32 cannot activate the FAS ACP of *E. coli*, even though its cognate ACP is an efficient substrate for the *E. coli* FAS ACPS. The viability of HT253 carrying *SVTN_RS23020* in non-permissive medium suggested that *SVTN_RS23020* encodes indeed the FAS ACPS in *S. vietnamensis*.

### Transcriptome analysis of the Δ*gra-orf32* mutant

As discussed earlier, some latent PPTase activity or crosstalk between pathways could possibly account for both the trace production in liquid YEME and the elevated production on ISP2 plates for granaticins. Analysis of the genomic sequence of *S. vietnamensis* GIMV4.0001 revealed 12 putative discrete PPTase genes in total, including two ACPS-type and ten Sfp-type genes (Supplementary Table S3). All except the FAS ACPS gene (*SVTN_RS23020*) were predicted to sit in the biosynthetic gene clusters of secondary metabolites. In the antiSMASH database (Blin et al., 2021), *S. vietnamensis* GIMV4.0001 was predicted to possess 30 biosynthetic clusters for secondary metabolites. For possible crosstalk in this case, it is more likely to occur between similar pathways. Besides the *gra* cluster, there are two additional biosynthetic gene clusters containing type II PKS (Supplementary Figure S11). To obtain more clues, a transcriptome analysis was carried out to assess the gene transcription activities at the genomic scale.

**FIGURE 3:**
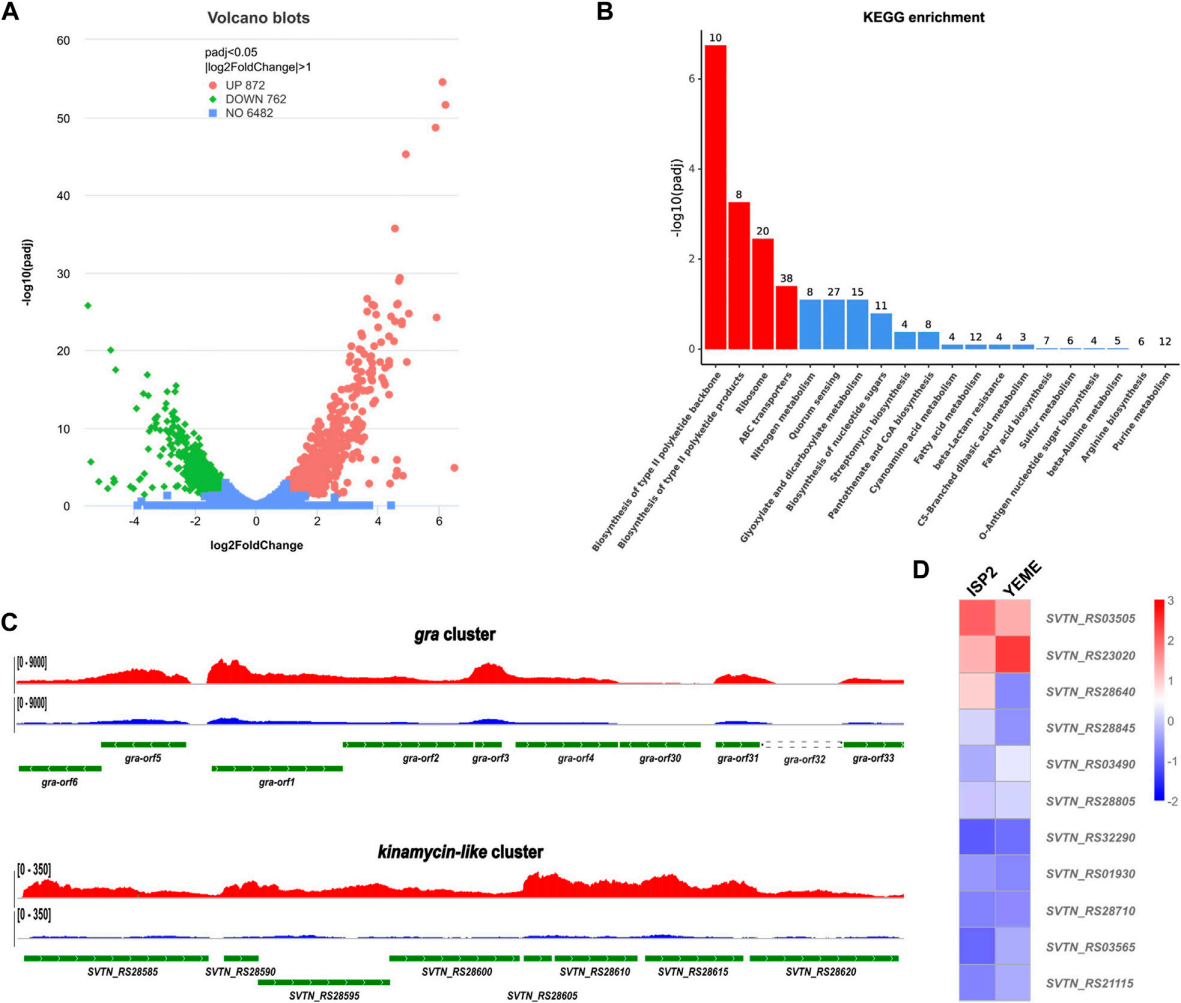
Comparative transcriptome analysis of the in-frame deletion mutant Δ*gra-orf32*. **(A)** Volcano blots showing differentially expressed genes (DEGs). Dispersion graph of the -log10(P adjusted) (*y-axis*) against the log2(FoldChange) (*x-axis*). Green and red dots represent genes that were significantly downregulated and upregulated, respectively, on ISP2 plates at the time point of 24 h, compared to those on YEME plates. **(B)** KEGG pathway enrichment analysis. Four out of the top 20 enriched pathways with significancy were identified (columns in red). The top two enriched pathways were type II pathways, namely, the granaticin biosynthetic pathway and the kinamycin-like biosynthetic pathway. The genes enriched into these two pathways were all significantly upregulated. **(C)** Visualization of RNA-seq across the core parts of the gene clusters of the enriched type II pathways. The read coverage counts for samples from ISP2 and YEME plates were colored in red and in blue, respectively. **(D)** Differential expression heatmap of the PPTase genes of *S. vietnamensis*.

After cultivation on ISP2 plate for 24 h, The mutant Δ*gra-orf32* cells start to produce the blue pigment, but no evident aerial hyphae and sporulation can be observed at this stage. Thus, this time point was chosen to collect the cells for total RNA preparation. The RNA-seq results showed that the transcription levels of more than 1,600 genes were significantly changed (*p* < 0.05, log2 fold change >1). Amongst them, 872 genes were upregulated and 762 genes were downregulated on ISP2 plates, compared to those on YEME plates (Figure 3A). KEGG pathway enrichment analysis showed that four pathways were enriched significantly, including two type II PKS biosynthesis pathways (Figure 3B), namely, the granaticin biosynthetic pathway and the kinamycin-like biosynthetic pathway. Genes enriched into these two pathways were significantly upregulated on ISP2 plates and most of them are the core PKS genes (Figure 3C). These results suggested that the kinamycin-like biosynthetic pathway was activated on ISP2 plates and might associate with the elevated production of granaticins.

Next, we inspected the expression pattern differences of all the predicted PPTase genes on both ISP2 and YEME plates. The result showed that most of the PPTase genes kept in low-levels of expression or inactivated, even the expression level of the FAS ACPS gene (*SVTN_RS23020*) was not as high as expected. Two PPTase genes, *SVTN_RS03505* and *SVTN_RS28640* were slightly upregulated on ISP2 plate, while the FAS ACPS gene (*SVTN_RS23020*) was downregulated, compared to those on YEME plates (Figure 3D). It is not surprising that *SVTN_RS28640* was upregulated since it locates in the kinamycin-like biosynthetic cluster. It is also understandable that the FAS ACPS gene (*SVTN_RS23020*) was downregulated since ISP2 plates are usually used for sporulation while *S. vietnamensis* is more inclined to vegetative growth on YEME plates, which means that more fatty acids are required to support the formation of cell membrane.

### Crosstalk led to the elevated granaticins production in the Δ*gra-orf32* mutant on solid ISP2 medium

**FIGURE 4:**
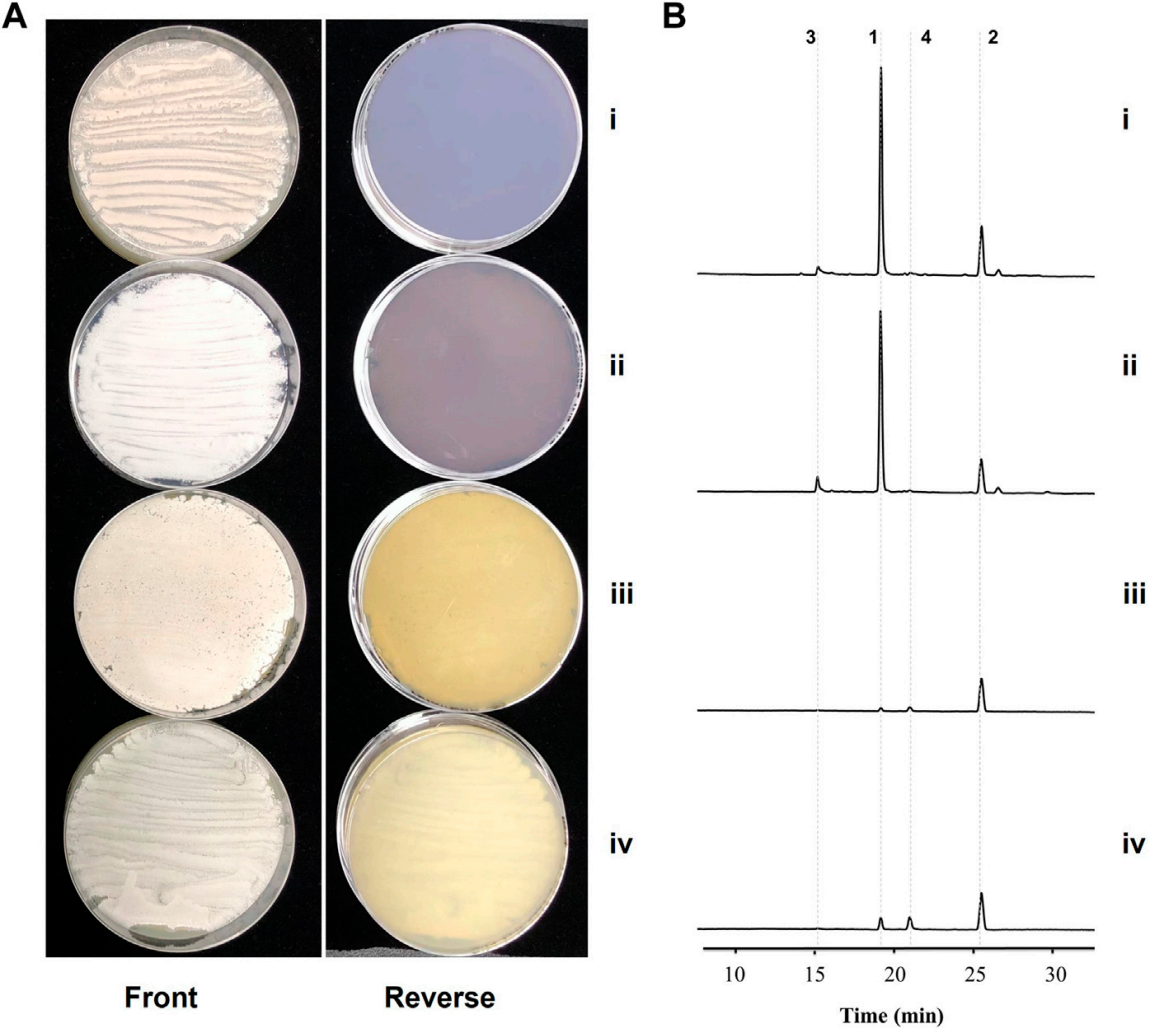
Inactivations of the spore pigment and the kinamycin-like pathways in the *gra-orf32* deletion background. i, the parent mutant Δ*gra-orf32*; ii, the double deletion mutant Δ*gra-orf32*Δ*pig-pks*; iii, the double deletion mutant Δ*gra-orf32*Δ*kina-like-pks*; iv, the triple deletion mutant Δ*gra-orf32*Δ*kina-like-pks*Δ*pig-pks*. **(A)** Cultures of mutant on ISP2 plates at 7 d **(B)** HPLC metabolic profiling of the mutants. The UV absorbance data were collected at the wavelength of 520 nm. **1**, granaticin; **2**, granaticin B; **3**, granaticinic acid; **4**, granaticinic acid B.

As implicated by the transcriptome analysis, a kinamycin-like biosynthetic pathway might be involved in the elevated production of granaticins in mutant Δ*gra-orf32*. Therefore, we set out to inactivate this pathway by in-frame deletion of the minimal PKS genes in the Δ*gra-orf32* mutant (Supplementary Figure S12). The production level of granaticins of the resulting mutant Δ*gra-orf32*Δ*kina-like-pks* decreased dramatically, only a similar trace amount of granaticins to those when cultivated in liquid YEME could be detected by HPLC (Figure 4), suggesting that the crosstalk between the granaticin and the kinamycin-like biosynthetic pathways should account for the elevated granaticins production in the Δ*gra-orf32* mutant.

To further explore any involvement of the spore pigment cluster (the other additional type II PKS) in the elevated production of granaticins in mutant Δ*gra-orf32*, the minimal PKS genes of the spore pigment was also deleted in the Δ*gra-orf32* mutant in a similar way (Supplementary Figure S13). The resulting mutant Δ*gra-orf32*Δ*pig-pks* did not change the production level of granaticins on ISP2, compared to the parent mutant Δ*gra-orf32* (Figure 4). The minimal PKS genes of the spore pigment was further deleted in the Δ*gra-orf32*Δ*kina-like-pks* double-deletion mutant to generate Δ*gra-orf32*Δ*kina-like-pks*Δ*pig-pks* (Supplementary Figure S14). This triple-deletion mutant still retained trace production of granaticins on ISP2 plate, which was similar to that of its parent strain Δ*gra-orf32*Δ*kina-like-pks* (Figure 4). These results were consistent with the transcriptome analysis and suggested that the spore pigment pathway was not involved in the granaticins production of the mutant Δ*gra-orf32*. The trace amount of production of granaticins thus might result from a latent PPTase activity.

### Overexpression of endogenous PPTase genes in the Δ*gra-orf32* mutant

**FIGURE 5:**
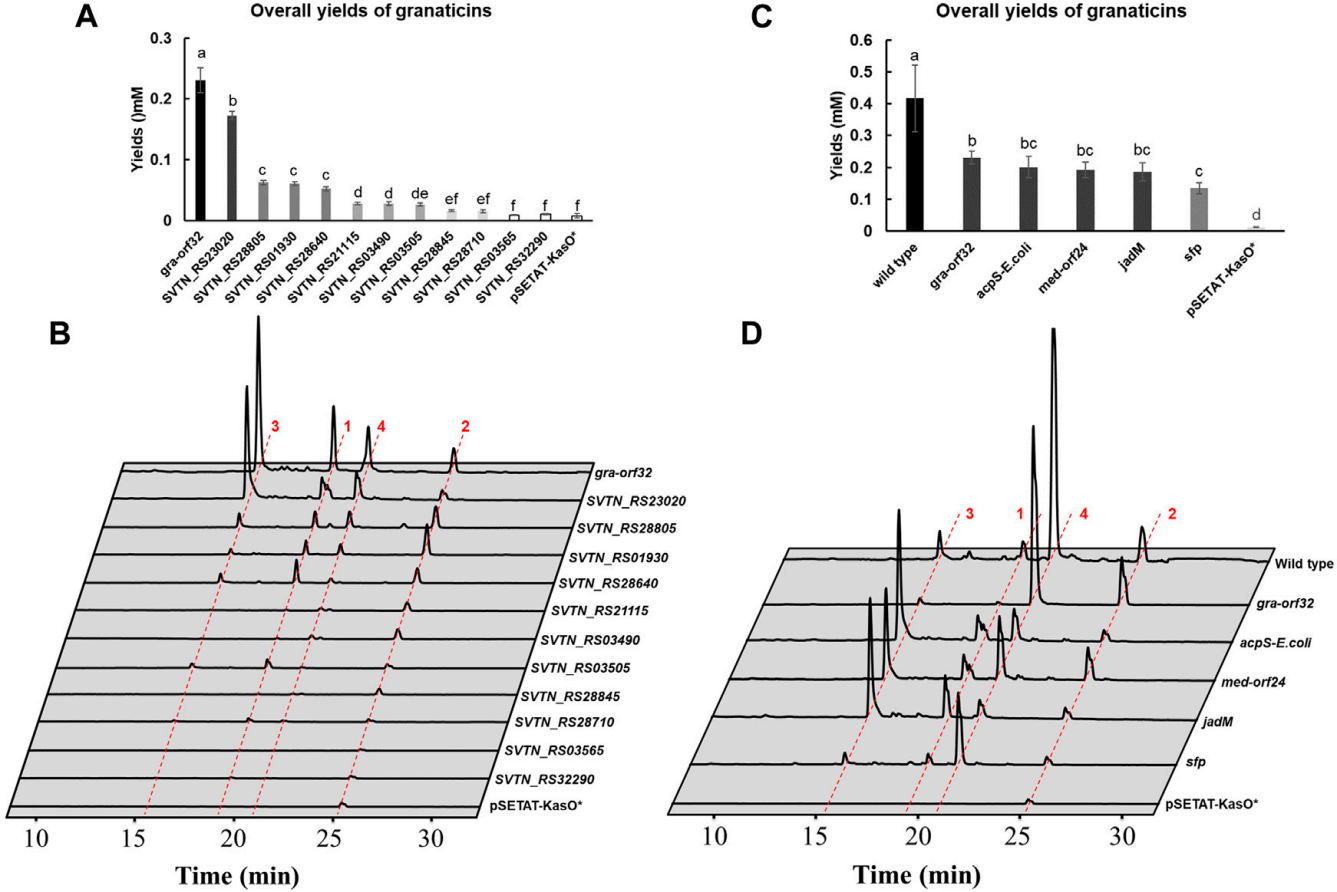
Overexpression of endogenous and exogenous PPTase genes in the mutant Δ*gra-orf32*. **1**, granaticin; **2**, granaticin B; **3**, granaticinic acid; **4**, granaticinic acid B. The UV absorbance data were collected at the wavelength of 520 nm. Statistical significances of quantifications were tested at *p* < 0.05 and indicated on top of the columns. **(A)** Quantifications of the mutant Δ*gra-orf32* carrying an additional copy of different endogenous PPTase genes under the strong promoter KasOp*. **(B)** HPLC metabolic profiling of the mutant Δ*gra-orf32* with overexpression of different endogenous PPTase genes. **(C)** Quantifications of the mutant Δ*gra-orf32* with overexpression of selected exogenous PPTase genes. **(D)** HPLC metabolic profiling of the mutant Δ*gra-orf32* with overexpression of selected exogenous PPTase genes.

With the results at hand, the trace production of granaticins of the Δ*gra-orf32* mutant was likely due to latent activities from other endogenous PPTases. And this mutant only produces a trace amount of granaticins, making it an excellent platform to probe the possible latent activities for the graACP from other endogenous PPTases. Additional copy of each of the eleven endogenous PPTase genes under the strong promoter KasOp* was individually introduced to Δ*gra-orf32* mutant using plasmid pSETAT-KasOp* as the delivering vector (Supplementary Figure S10). As mentioned earlier, the complementation strain Δ*gra-orf32*:*gra-orf32* could only recover 35–55% production of granaticins. Therefore, this complementation strain was used for the comparative analysis since these endogenous PPTase genes were introduced based on the same vector and under the same promoter as in the complementation experiment. Surprisingly, nine out of 11 endogenous non-cognate PPTases could activate the granaticins production to varied extents (Figures 5A,B). Amongst them, the FAS ACPS (SVTN_RS23020) was top-ranked, with about 75% overall yield of granaticins to that of complementation strain. Three Sfp-type PPTases (SVTN_RS28805, SVTN_RS01930 and SVTN_RS28640) showed comparable activities of activation (ranging from 23%–27%). Another ACPS-type PPTase (SVTN_RS21115) and two Sfp-type PPTases (SVTN_RS03490 and SVTN_RS03505) shared similar but much weaker activities of activation (11.4%–12%). SVTN_RS28845 and SVTN_RS28710 showed marginal activities while no evident activations of the remaining two PPTases (SVTN_RS03565 and SVTN_RS32290) were observed. These results indicated that most of the endogenous PPTases could catalyze the PPant transfer to graACP with varied efficiencies, of which the FAS ACPS (SVTN_RS23020) were the most efficient. Thus, the trace production of granaticins might be mainly attributed to the latent activity from the FAS ACPS (SVTN_RS23020).

### Exogenous PPTases of both the Sfp type and the ACPS type could activate the production of granaticins

A dozen of characterized type II PKS clusters contain a putative PPTase gene, including jadomycin (*jadM*), griseorhodin (*grhF*), medermycin (*med-orf24*) and oviedomycin (*ovmF*). To investigate whether these in-cluster Sfp-type PPTases can recognize the ACPs of other type II PKS pathways, *jadM* and *med-orf24*, together with the prototype genes, *sfp* from *B. substilis* and *acpS* from *E. coli*, were chosen to be overexpressed in the mutant Δ*gra-orf32* (Supplementary Figure S15). The ACPS and Sfp enzymes were reported active *in vitro* for the graACP or with broad substrate promiscuity (Gehring et al., 1997; Quadri et al., 1998). HPLC analysis showed that Δ*gra-orf32* carrying *jadM* or *med-orf24* could restore production of granaticins at levels of 83% and 80% to that of the complementation strain (Figures 5C,D), respectively, suggesting that dedicated PPTases of type II PKSs could efficiently activate ACPs of other type II PKS pathways. Not surprisingly, introduction of either *sfp* or *acpS* genes could also activate the granaticins production. The production of the strain with the *E. coli acpS* gene reached an astonishing production level of 87% to that of the complementation strain (Figures 5C,D), yet this result was consistent with the early *in vitro* study (Gehring et al., 1997).

## Discussion

The ability of the *E. coli* FAS ACPS to *in vitro* activate ACPs of several type II PKS reported by early studies (Carreras et al., 1997; Cox et al., 1997; Gehring et al., 1997) and observations of lacks of apparent PPTase genes in many type II polyketide clusters led a wide acknowledgement that ACPs of type II PKSs are usually activated by the hosts’ FAS ACPSs. However, a number of aromatic polyketides, such as granaticin, medermycin, jadomycin, oviedomycin, griseorhodin and landomycin have been found to harbour a putative Sfp-type PPTase gene in their biosynthetic clusters (Ichinose et al., 1998; Westrich et al., 1999; Wang et al., 2001; Li and Piel, 2002; Ichinose et al., 2003; Lombó et al., 2004). Disruption of *jadM*, the putative PPTase gene in the jadomycin biosynthetic cluster in *S. venezuelae* ISP5230 caused more than 95% loss of jadomycin production (Wang et al., 2001). FdmW is the first characterized in-cluster PPTase of ACPS type (Huang et al., 2006). Inactivation of *fdmW* resulted in ∼93% reduction of fredericamycin production in *S. griseus*, and FdmW preferred its cognate ACP *in vitro* in spite of its ability to phosphopantetheinylate various ACPs, making the authors to conclude FdmW a dedicated PPTase for fredericamycin biosynthesis. A recent study showed that a stand-alone Sfp-type PPTase is required for activation of the ACP of xanthomonadin which is biosynthesized by a type II PKS (Chen et al., 2022). While preparing the manuscript, a newly online study reported another in-cluster Sfp-type PPTase required for production of the aromatic polyketide rishirilide in *S. xanthophaeus* no2 (Zhang et al., 2022). In our study, inactivation and complementation of *gra-orf32* and overexpression of other characterized PPTase genes in *S. vietnamensis* demonstrated that Gra-ORF32 is a dedicated PPTase for granaticin biosynthesis. Further search against the database revealed that more than 50 uncharacterized type II PKSs harbor Sfp-type PPTase genes (Supplementary Figure S7). These results and together with previously reported studies (Wang et al., 2001; Huang et al., 2006; Chen et al., 2022; Zhang et al., 2022) suggested that many ACPs of type II PKSs require a specific PPTase rather than the FAS ACPS of the host for efficient phosphopantetheinylation *in vivo*. Some type II PKS pathways have evolved to accommodate a dedicated PPTase gene in their biosynthetic clusters while the others recruit a specific non-in-cluster Sfp-type PPTase for activation.

The Sfp-type PPTases exhibit a pseudodimeric fold with two structurally similar subdomains connected by a short polypeptide loop and are approximately twice of ACPS-type enzymes in size (∼230 *v. s.* ∼120 amino acids), which suggests that they evolved by gene duplication from an ACPS ancestor (Sánchez et al., 2001). Gra-ORF32 was initially predicted to consist of 218 amino acids, but this protein was dysfunctional in the complementation experiment. An extended version of the protein with 38 additional amino acids at the N terminus was proved to be active, suggesting that this additional Arg- and Pro-rich N terminus is necessary for activity. Similar situation was also reported in FdmW (Huang et al., 2006). Sequence alignment revealed that all cluster-situated PPTases of characterized type II PKSs possess this additional Arg- and Pro-rich N terminus (Supplementary Figure S16), indicating that it may be a common characteristic for type-II-PKS-dedicated PPTases and may play an important structural or functional role during the interactions of PPTases with the substrate ACPs. Although both ACPS-type and Sfp-type PPTases have been found to possess relatively broad substrate specificity (Beld et al., 2014), the “ancestral” ACPSs are usually involved in essential fatty acid biosynthesis while the “descendant” Sfp-type PPTases are involved in non-essential functions under normal conditions, such as biosynthesis of secondary metabolites. To test whether the *E. coli* FAS ACP whose cognate ACPS can efficiently activate the graACP is an effective substrate of the graACP’s cognate PPTase, we overexpressed *gra-orf32* in a conditionally ACPS-deficient *E. coli* mutant HT253. However, Gra-ORF32 could not functionally compensate *E. coli* FAS ACPS, which suggested that this Sfp-type PPTase has evolved to be functionally segregated from fatty acid biosynthesis.

*Streptomyces* species usually have potentials to produce more than twenty or even thirty secondary metabolites, many of which need one or more carrier protein(s) for biosynthesis. In contrast, the number of PPTases is typically less than 10, which is much less than that of carrier proteins, indicating that a PPTase can phosphopantetheinylate multiple carrier proteins. Previous study showed that a complicated interaction network between PPTases and their substrate carrier proteins exist in *S. tsukubaensis* L19, where PPTases could phosphopantetheinylate multiple carrier proteins and carrier proteins could also be activated by multiple PPTases (Wang et al., 2016). In the genome of *S. vietnamensis* GIMV4.0001, twelve discrete PPTase genes were predicted in total (Supplementary Table S3), which is much more than previously studied strains. A phylogenetic tree built with 292 entities showed that these PPTases were scattered across the tree (Supplementary Figure S17). We overexpressed all the eleven non-cognate PPTase genes in the mutant Δ*gra-orf32*. Although the graACP has its cognate PPTase, to our surprise, nine out of eleven non-cognate PPTases, including two ACPS and seven Sfp-type PPTases, could activate the graACP to produce granaticins to varied extents under the test condition. This indicated that ACPs of type II PKSs could also be widely recognized as effective substrates by phylogenetically distant Sfp-type PPTases.

Because ease of genetic manipulations and fast growth, researchers have showed strong interests to functionally reconstitute type II PKSs in *E. coli* (Park et al., 2020). However, *E. coli* is not a native polyketide producer, hence production of aromatic polyketides in *E. coli* remains challenging (Gao et al., 2021). One of the problems is the failure of phosphopantetheinylation of the ACPs from type II PKSs (Gao et al., 2021). Co-expression of a PPTase-encoding gene with the interested cluster is an effective strategy to overcome such issue, thus PPTases with broad substrate specificities are desperately needed. However, the well-known promiscuous PPTase, Sfp, was found incapable of phosphopantetheinylation of the ACP of kinamycin in *E. coli* (Liu et al., 2020), suggesting that Sfp has its limitations for activating ACPs of type II PKSs. In the current study, overexpression of both *E. coli* FAS *acpS* and the in-cluster PPTase genes of type II PKSs, *med-orf24* and *jadM*, could enable the Δ*gra-orf32* mutant to restore the granaticins production at levels of over 80% to that of the native PPTase-encoding *gra-orf32*. Med-ORF24 and JadM were clustered in different clades with Gra-ORF32 in the phylogenetic tree (Supplementary Figure S17), only sharing 25.4% and 22.3% identities with Gra-ORF32, respectively. This indicated that the phylogenetically distant in-cluster PPTases have potentials of broad substrate specificities to the ACPs of type II PKSs. Amongst the tested exogenous PPTases, the *E. coli* FAS ACPS showed the highest activity to activate the granaticins production. This result was consistent with the previous report, where co-expression of the *E. coli* FAS ACPS gene with the ACP genes of actinorhodin and griseusin in *E. coli* leading to high levels of production of active *holo*-ACPs (Cox et al., 1997). The results present here suggested that co-expression of the *E. coli* FAS *acpS* or an in-cluster PPTase-encoding gene of type II PKSs could efficiently activate the ACPs of the interested type II PKSs, which would facilitate the future functional reconstitutions of type II PKSs in *E. coli*.

Since the carrier proteins keep in inactive state until they are phosphopantetheinylated by PPTases, it is not surprising that PPTases serve as regulatory checkpoints, through post-translational modications, for biosynthesis of both the primary fatty acid and the secondary metabolites. A PPTase-based activation strategy has been developed to awaken the silent biosynthetic pathways of secondary metabolites (Zhang et al., 2017). However, the regulatory effects on the biosynthesis of secondary metabolites seems complicated. NysF, a Sfp-type PPTase, was reported to negatively regulate the nystatin biosynthesis (Volokhan et al., 2005), while overexpression of two promiscuous PPTase genes, *sfp* and *svp* in *S. alboniger* NRRL B-1832 led to activation of the production of puromycins whose biosynthesis doesn’t require the involvement of carrier protein (Yan et al., 2018). In the current study, although overexpression of both the FAS *acpS* gene of *S. vietnamensis* and most of other endogenous PPTase genes in the Δ*gra-orf32* mutant could activate the granaticins production, deletion of *gra-orf32* resulted in trace production of granaticins, which clearly showed that the activities of PPTases were strictly regulated. By combining the transcriptome data with the overexpression results, regulations at transcription level can be easily identified since most PPTase genes were transcriptionally low-active or inactive under normal physiological conditions while overexpression of these endogenous PPTase genes led to significant activation of the granaticins production in the Δ*gra-orf32* mutant. However, regulation at the transcriptional level is not sufficient to explain what we have observed. Transcriptome analysis showed that the transcription level of *SVTN_RS23020* was top-ranked amongst all endogenous PPTase genes in the Δ*gra-orf32* mutant (Figure 3D), which is consistent with its crucial role in the primary metabolism. Thus, one could envisage that a quantity of the SVTN_RS23020 protein enough to support the growth constantly exists in the cell until at least the plateau growth stage. Previous *in vitro* activation of the graACP by the *E. coli* ACPS (Gehring et al., 1997) and *in vivo* activation of the granaticins production in the Δ*gra-orf32* mutant by overexpressing the FAS ACPS gene (*SVTN_RS23020*) of *S. vietnamensis* in the current study indicated that the graACP is an efficient substrate for a variety of FAS ACPSs, which means that the graACP is a competing substrate for the FAS ACPS of *S. vietnamensis*. However, the Δ*gra-orf32* mutant only produced a trace amount of granaticins, suggesting that the catalytic activity of the FAS ACPS (SVTN_RS23020) was almost segregated from the graACP under natural physiological conditions. The potential spatial-temporal expression differences might contribute to, but could not achieve such activity segregation alone since the similar situation happened in the biosynthesis of the closely related actinorhodin, in which the FAS ACPS (SCO4744) of *S. coelicolor* could efficiently activate the ACPs of both fatty acid and actinorhodin *in vivo* (Cox et al., 2002). Regulation at the protein-protein interaction level might be an alternative explanation. However, when only the competition between the ACP substrates is considered, a similar unsatisfactory situation emerges. The biosynthesis of actinorhodin can be efficiently activated without a dedicated PPTase, while the biosynthesis of granaticin cannot. The differences in substrate competitiveness may contribute to, but cannot make such activity disparity alone since, as mentioned above, the ACPs of granaticin and actinorhodin are both efficient substrates for the FAS ACPSs of their own hosts. A recent structural study showed that the *E. coli* ACPS and its *holo*-ACP product can form hexamers with 3:3 stoichiometry and the ACPS binds the *holo*-ACP product much more tightly than the substrate *apo*-ACP, leading the authors to hypothesize that fatty acid biosynthesis can be regulated by interaction between the ACPSs with their *holo*-ACP products (Marcella et al., 2017). Our observations favored this hypothesis. If the FAS ACPS binds the *holo*-ACP product with much higher affinity in *S. vietnamensis*, the probability of binding the graACP for the FAS ACPS may decline exponentially, which will result in trace production of granaticins in the Δ*gra-orf32* mutant.

Crosstalk between pathways has been widely reported at both regulatory and structural genes levels (Cano-Prieto et al., 2015; Chater, 2016; Losada et al., 2017). Crosstalk between the exogenous biosynthetic gene cluster and the host native pathway has also been reported (Zhao et al., 2016). In our study, the Δ*gra-orf32* mutant produced much more granaticins on ISP2 plates than on YEME plates. KEGG enrichment analysis of the RNA-seq data revealed that the *gra* pathway itself and a kinamycin-like type II PKS pathway were significantly enriched when this mutant was cultivated on ISP2 plates. Further gene disruption experiments suggested crosstalk between these two type II PKS pathways was the main reason for the elevated production of granaticins. However, whether crosstalk happed between the KS of granaticin and the ACP of the kinamycin-like pathway or between the downstream enzymes of granaticin and the KS of the kinamycin-like pathway remains to be investigated.

In summary, we identified Gra-ORF32 as an in-cluster PPTase that is dedicated for granaticin biosynthesis by gene disruption and complementation and overexpression of other characterized PPTase genes in *S. vietnamensis*, and the Arg- and Pro-rich N terminus was found to be crucial for the catalytic activity. This specific PPTase is inactive for the FAS ACP, indicating that it has evolved to be functionally segregated from fatty acid biosynthesis. Overexpression of endogenous PPTase genes indicated that ACPs of type II PKSs could be widely recognized as effective substrates by phylogenetically distant Sfp-type PPTases. Both the FAS ACPSs and homologous in-cluster PPTases of type II PKSs were proved to be able to activate the ACP of granaticin *in vivo* with high efficiency, but only Gra-ORF32 could efficiently activate the granaticins production in the wild type strain under natural physiological conditions, indicating that the activity of the FAS ACPS was strictly regulated, possibly by binding the FAS *holo*-ACP product with high affinity. Our findings would contribute to a more comprehensive understanding of how the ACPs of type II PKSs are activated and facilitate the future functional reconstitutions of type II PKSs in *E. coli*.

## Data Availability

The raw data for RNA-seq were deposited into the NCBI GEO database under the accession number GSE219231. Other datasets generated for this study are included in the article/Supplementary Material.
