# Corrigendum: An in-cluster Sfp-type phosphopantetheinyl transferase instead of the *holo*-ACP synthase activates the granaticin biosynthesis under natural physiological conditions

**DOI:** 10.3389/fchem.2024.1443723

**Published:** 2024-06-18

**Authors:** Ming-Rong Deng, Sin Yu Chik, Yan Li, Honghui Zhu

**Affiliations:** Key Laboratory of Agricultural Microbiomics and Precision Application (MARA), Guangdong Provincial Key Laboratory of Microbial Culture Collection and Application, Key Laboratory of Agricultural Microbiome (MARA), State Key Laboratory of Applied Microbiology Southern China, Institute of Microbiology, Guangdong Academy of Sciences, Guangzhou, China

**Keywords:** phosphopantetheinyl transferase, granaticin, type II polyketide synthase, *gra-orf32*, acyl carrier protein, crosstalk, *Streptomyces vietnamensis*

In the published article, there was an error in the **Funding** statement. The grant number of the National Natural Science Foundation of China was incorrect. The correct statement appears below:

“This work was financially supported in part by the National Natural Science Foundation of China (Grant No. 32070047) and the GDAS Special Project of Science and Technology Development (2020GDASYL-20200301003).”

The authors apologize for this error and state that this does not change the scientific conclusions of the article in any way. The original article has been updated.

