# Supplementary material for: An in-cluster Sfp-type phosphopantetheinyl transferase instead of the holo-ACP synthase activates the granaticin biosynthesis under natural physiological conditions
Source: Front Chem. 2022 Dec 22;10:1112362. doi: 10.3389/fchem.2022.1112362 (PMC9813960; doi:10.3389/fchem.2022.1112362)
Supplement: Supplementary file 1 [file DataSheet1.pdf]

Supplementary Material

An in-cluster Sfp-type phosphopantetheinyl transferase instead of the *holo*-ACP synthase activates the granaticin biosynthesis under natural physiological conditions

Ming-Rong Deng*, Sin Yu Chik, Yan Li, Honghui Zhu*

Key Laboratory of Agricultural Microbiomics and Precision Application (MARA), Guangdong Provincial Key Laboratory of Microbial Culture Collection and Application, Key Laboratory of Agricultural Microbiome (MARA), State Key Laboratory of Applied Microbiology Southern China, Institute of Microbiology, Guangdong Academy of Sciences, Guangzhou, People's Republic of China

*** Correspondence:**
Ming-Rong Deng
dengmr@gdim.cn
Honghui Zhu
zhuhh_gdim@163.com

Supplementary Table S1. Strains and plasmids used or generated in this study.

Strain/ plasmid	Description	Source (Reference)
**Strain**		
E. coli		
NEB turbo	*E. coli* host for general cloning	New England Biolabs
ET12567/pUZ8002	Methylation-deficient *E. coli* host for intergeneric conjugation	(MacNeil et al., 1992)
HT253	W3110 pdxJ8::ΔTn10	(Takiff et al., 1992)
HT253/pET28b+	HT253 carrying the blank plasmid pET28b(+) as a control	This study
HT253/*gra-orf32*	HT253 carrying the recombinant plasmid pET28b-gra-orf32	This study
HT253/*SVTN_RS23020*	HT253 carrying the recombinant plasmid pET28b-SVTN_RS23020	This study
HT253/*SVTN_RS21115*	HT253 carrying the recombinant plasmid pET28b-SVTN_RS21115	This study
Streptomyces		
S. vietnamensis GIMV4.0001	wild type strain	(Zhu et al., 2007)
Δ*gra-orf32*	in-frame deletion of *gra-orf32*	This study
Δ*gra-orf32*::*gra-orf32*	the complementary strain of the Δ*gra-orf32* mutant	This study
Δ*gra-orf32*Δ*pig-pks*	in-frame deletion of the minimal PKS genes (*ks*, *clf* and *acp*) of the predicted spore pigment in the genetic background of *gra-orf32* deletion	This study
Δ*gra-orf32*Δ*kina-like-pks*	in-frame deletion of the minimal PKS genes (*ks*, *clf* and *acp*) of the predicted kinamycin-like cluster in the genetic background of *gra-orf32* deletion	This study
Δ*gra-orf32*Δ*kina-like-pks*Δ*pig-pks*	in-frame deletion of the minimal PKS genes (*ks*, *clf* and *acp*) of the predicted spore pigment using Δ*gra-orf32*Δ*kina-like-pks* as a parent strain	This study
Δ*gra-orf32*::*pSETAT-KasOp**	Δ*gra-orf32* mutant integrated with the blank vector pSETAT-KasOp* as control	This study
Δ*gra-orf32*::*SVTN_RS01930*	Δ*gra-orf32* mutant with overexpression of *SVTN_RS01930*	This study
Δ*gra-orf32*::*SVTN_RS03490*	Δ*gra-orf32* mutant with overexpression of *SVTN_RS03490*	This study
Δ*gra-orf32*::*SVTN_RS03505*	Δ*gra-orf32* mutant with overexpression of *SVTN_RS03505*	This study

Δ*gra-orf32*::*SVTN_RS03565*	Δ*gra-orf32* mutant with overexpression of *SVTN_RS03565*	This study
Δ*gra-orf32*::*SVTN_RS21115*	Δ*gra-orf32* mutant with overexpression of *SVTN_RS21115*	This study
Δ*gra-orf32*::*SVTN_RS23020*	Δ*gra-orf32* mutant with overexpression of *SVTN_RS23020*	This study
Δ*gra-orf32*::*SVTN_RS28640*	Δ*gra-orf32* mutant with overexpression of *SVTN_RS28640*	This study
Δ*gra-orf32*::*SVTN_RS28710*	Δ*gra-orf32* mutant with overexpression of *SVTN_RS28710*	This study
Δ*gra-orf32*::*SVTN_RS28805*	Δ*gra-orf32* mutant with overexpression of *SVTN_RS28805*	This study
Δ*gra-orf32*::*SVTN_RS28845*	Δ*gra-orf32* mutant with overexpression of *SVTN_RS28845*	This study
Δ*gra-orf32*::*SVTN_RS32290*	Δ*gra-orf32* mutant with overexpression of *SVTN_RS32290*	This study
Δ*gra-orf32*::*acpS-E.coli*	Δ*gra-orf32* mutant with overexpression of the acyl carrier protein synthase gene *acpS* from the fatty acid biosynthetic pathway of *E. coli*	This study
Δ*gra-orf32*::*sfp-B.subtilis*	Δ*gra-orf32* mutant with overexpression of the pptase gene *sfp* from the surfactin biosynthetic pathway of *Bacillus subtilis*	This study
Δ*gra-orf32*::*jadM*	Δ*gra-orf32* mutant with overexpression of the predicted pptase gene *jadM* from the jadomycin biosynthetic pathway	This study
Δ*gra-orf32*::*med-orf24*	Δ*gra-orf32* mutant with overexpression of the predicted pptase gene *med-orf24* from the medermycin biosynthetic pathway	This study
**Plasmid**		
pKC1139	Apr^r, temperature sensitive vector used for construction of the disruption plasmid of the *gra-orf32* gene	(Bierman et al., 1992)
pYH7	Apr^r, vector for construction of the disruption plasmids of the predicted kinamycin-like and spore-pigment minimal PKS genes	(Sun et al., 2006)
pSETAT-KasOp*	Amp^r, $Thio^r$, pSET152-based and derived from the plasmid pSET-KasOp*, containing the promoter KasOp*	(Pan et al., 2017)
pKC-Δgra-orf32	Apr^r, in-frame deletion plasmid used for construction of the Δ*gra-orf32* mutant	This study
pYH-Δpig-pks	Apr^r, in-frame deletion plasmid used for construction of the Δ*gra-orf32*Δ*pig-pks* mutant	This study

pYH-Δkina-like-pks	Apr^r, in-frame deletion plasmid used for construction of the Δ*gra-orf32*Δ*kina-like-pks* mutant	This study
pSETAT-gra-orf32	Amp^r, $Thio^r$, complementary plasmid used for re-introduction of *gra-orf32* into the Δ*gra-orf32* mutant	This study
pSETAT-SVTN_RS01930	Amp^r, $Thio^r$, pSETAT-KasOp*-based plasmid used for overexpression of *SVTN_RS01930* in the Δ*gra-orf32* mutant	This study
pSETAT-SVTN_RS03490	Amp^r, $Thio^r$, pSETAT-KasOp*-based plasmid used for overexpression of *SVTN_RS03490* in the Δ*gra-orf32* mutant	This study
pSETAT-SVTN_RS03505	Amp^r, $Thio^r$, pSETAT-KasOp*-based plasmid used for overexpression of *SVTN_RS03505* in the Δ*gra-orf32* mutant	This study
pSETAT-SVTN_RS03565	Amp^r, $Thio^r$, pSETAT-KasOp*-based plasmid used for overexpression of *SVTN_RS03565* in the Δ*gra-orf32* mutant	This study
pSETAT-SVTN_RS21115	Amp^r, $Thio^r$, pSETAT-KasOp*-based plasmid used for overexpression of *SVTN_RS21115* in the Δ*gra-orf32* mutant	This study
pSETAT-SVTN_RS23020	Amp^r, $Thio^r$, pSETAT-KasOp*-based plasmid used for overexpression of *SVTN_RS23020* in the Δ*gra-orf32* mutant	This study
pSETAT-SVTN_RS28640	Amp^r, $Thio^r$, pSETAT-KasOp*-based plasmid used for overexpression of *SVTN_RS01930* in the Δ*gra-orf32* mutant	This study
pSETAT-SVTN_RS28710	Amp^r, $Thio^r$, pSETAT-KasOp*-based plasmid used for overexpression of *SVTN_RS28710* in the Δ*gra-orf32* mutant	This study
pSETAT-SVTN_RS28805	Amp^r, $Thio^r$, pSETAT-KasOp*-based plasmid used for overexpression of *SVTN_RS28805* in the Δ*gra-orf32* mutant	This study
pSETAT-SVTN_RS28845	Amp^r, $Thio^r$, pSETAT-KasOp*-based plasmid used for overexpression of *SVTN_RS28845* in the Δ*gra-orf32* mutant	This study
pSETAT-SVTN_RS32290	Amp^r, $Thio^r$, pSETAT-KasOp*-based plasmid used for overexpression of *SVTN_RS32290* in the Δ*gra-orf32* mutant	This study
pSETAT-acpS-Ec	Amp^r, $Thio^r$, pSETAT-KasOp*-based plasmid used for overexpression of the *holo* acyl carrier protein synthase gene *acpS* from the fatty acid biosynthetic pathway of *E. coli* in the Δ*gra-orf32* mutant	This study

pSETAT-sfp-Bs	Amp^r, $Thio^r$, pSETAT-KasOp*-based plasmid used for overexpression of the pptase gene *sfp* from the surfactin biosynthetic pathway of *Bacillus subtilis* in the Δ*gra-orf32* mutant	synthesized by Azenta China
pSETAT-jadM	Amp^r, $Thio^r$, pSETAT-KasOp*-based plasmid used for overexpression of the predicted pptase gene *jadM* from jadomycin biosynthetic pathway in the Δ*gra-orf32* mutant	synthesized by Azenta China
pSETAT-med-orf24	Amp^r, $Thio^r$, pSETAT-KasOp*-based plasmid used for overexpression of the predicted pptase gene *med-orf24* from medermycin biosynthetic pathway in the Δ*gra-orf32* mutant	synthesized by Azenta China
pET28b (+)	Km^r, vector for expression of N-terminally 6xHis-tagged proteins with T7 promoter	Novagen
pET28b-gra-orf32	Km^r, pET28b(+) carrying the *gra-orf32* gene	synthesized by Azenta China
pET28b-SVTN_RS23020	Km^r, pET28b(+) carrying the *SVTN_RS23020* gene	synthesized by Azenta China
pET28b-SVTN_RS21115	Km^r, pET28b(+) carrying the *SVTN_RS21115* gene	synthesized by Azenta China

Supplementary Table S2. Primers used in this study.

Primer	Nucleotide Sequence (5'-3')	Description
KC-orf32LF	taaaacgacggccagtgccaagcttGGAGTGGATCGCGTACAAGC	Used for amplification of the homologous arms for the Δ*gra-orf32* mutant construction
KC-orf32LR	catcgtcggcGTCCTCGACGGTGCCGTG	
KC-orf32RF	cgtcgaggacGCCGACGATGAGCCAGTTC	
KC-orf32RR	acagctatgacatgattacgaattcCTTCCGATGAACACCACCC	
YH-PigpksLF	tgatcaaggcgaatacttcaGGTTCACTCATCCGCCCCGTGGGACT	Used for amplification of the homologous arms for construction of the disruption plasmid pYH-Δpig-pks
YH-PigpksLR	cgttgttgacCGGCGGGTCACTCGAGGG	
YH-PigpksRF	tgacccgccgGTCAACAACGCCCTCGCAGGAGTCT	
YH-PigpksRR	cgtcgacctgcaggcatgcAAGACCGAACCGCGCACCCTGCGACGCCTGCTG	
YH-kinapksLF	tgatcaaggcgaatacttcaGATCAAGGCCGCCTTCGGTGGT	Used for amplification of the homologous arms for construction of the disruption plasmid pYH-Δkina-like-pks
YH-kinapksLR	aggcggcgcgAACCCGTCGCCCGGTCA	
YH-kinapksRF	gcgacgggttCGCGCCGCCTGAATCGTCTTGA	
YH-kinapksRR	cgtcgacctgcaggcatgcATGGTGCTCAGCGCGGAGTGCACGTACGCG	
COM-orf32F	cgtgcaggactgggggagttATGCGGACACCACGGGGCCGAG	Used for construction of the complementary plasmid pSETAT-gra-orf32
COM-orf32R	aattcacgtcatatgtcagactagTCATGGAACTGGCTCATCGTCGGCTGAG	
COM-RS01930F	cgtgcaggactgggggagttATGATCCCGCTGGTTGCCCC	Used for construction of the plasmid pSETAT-SVTN_RS01930
COM-RS01930R	agctcacgtcatatgTCAGTCACCGGCGGAGCGCCAC	
COM-RS03490F	cgtgcaggactgggggagttATGCTCGCCTCTCTGCTG	Used for construction of the plasmid pSETAT-SVTN_RS03490
COM-RS03490R	agctcacgtcatatgTCAGTCATGCCGGGACCAGCAC	
COM-RS03565F	cgtgcaggactgggggagttATGACCGGCATCGTCCTGGTG	Used for construction of

COM-RS03565R	agctcacgtcatatgTCAGTCACCGGCCGTGGGTGGAG	the plasmid pSETAT-SVTN_RS03565
COM-RS03505F<br>COM-RS03505R	cgtgcaggactgggggagttATGACCGTCCCCGGAGGCG<br>gagctcacgtcatatgTCAGCTACGCGGGCCCCAGGG	Used for construction of the plasmid pSETAT-SVTN_RS03505
COM-RS21115F<br>COM-RS21115R	cgtgcaggactgggggagttATGCCGGCCCGTCCCG<br>agctcacgtcatatgtcAGTCAGCTGTTCGATGAGGAAGGGGTG	Used for construction of the plasmid pSETAT-SVTN_RS21115
COM-RS23020F<br>COM-RS23020R	cgtgcaggactgggggagttATGATCATTGGGGTGGGGATCGA<br>agctcacgtcatatgtcAGTCAGCCCTCCGCGATCAC	Used for construction of the plasmid pSETAT-SVTN_RS23020
COM-RS28640F<br>COM-RS28640R	cgtgcaggactgggggagttATGCCCGCGGTGTCCACG<br>agctcacgtcatatgtcAGTCAGGTTCGACTGCCCGGGG	Used for construction of the plasmid pSETAT-SVTN_RS28640
COM-RS28710F<br>COM-RS28710R	cgtgcaggactgggggagttATGCCTGAGCTCGCACACG<br>agctcacgtcatatgtcAGTCAGCTCGCCGTGGCGAG	Used for construction of the plasmid pSETAT-SVTN_RS28710
COM-RS28805F<br>COM-RS28805R	cgtgcaggactgggggagttATGATCGAGGAACTGCTCCCGTC<br>agctcacgtcatatgtcAGTCAGGGAGCGGGGACCGC	Used for construction of the plasmid pSETAT-SVTN_R28805
COM-RS28845F<br>COM-RS28845R	cgtgcaggactgggggagttATGGCGCGCGCCGTGCCC<br>agctcacgtcatatgtcAGTCAGAGGTGCTCCTTCGGGGCCG	Used for construction of the plasmid pSETAT-SVTN_RS28845
COM-RS32290F<br>COM-RS32290R	cgtgcaggactgggggagttATGGACGCCGTGCTGGCG<br>agctcacgtcatatgtcAGTCAGGACGTTCGGGACGCCGAG	Used for construction of the plasmid pSETAT-SVTN_RS32290

152-psf-F<br>152-psf-R	CGCCAGGGTTTTCCCAGTCAC<br>TGCTTCCGGCTCGTATGTTGTG	Sequencing primers for pSETAT-KasOp* based plasmids
YH7seqF<br>YH7seqR	CGATCGAGGTCGGGGAGTCC<br>GAAGCAGGGTTATGCAGCGG	Sequencing primers for pYH7 based plasmids
V-psetat-F<br>V-psetat-R	GCGCAACTGTTGGGAAGGGC<br>CACTCATTAGGCACCCCAGGCTT	Used for PCR confirmation of the genotype of the Δ*gra-orf32* mutant carrying various pptase genes
V32LOF<br>V32RIR<br>V32LIF<br>V32ROR	CTGGAGATGGACACCGTCA<br>TTCCGGTCATGTGGGTCT<br>GGCGGCCGTTGCTCGTGCA<br>CGTTTTCCGCGGACCGACG	Used for PCR confirmation of the genotype of the Δ*gra-orf32* mutant
V-kinapks-OF1<br>V-kinapks-IF2<br>V-kinapks-IR3<br>V-kinapks-OR4	CGCCGCAGGCCATTCGTGAT<br>TCGAGAAGGCCAAGTCCGACCCG<br>TGGCCGAGGCGAGGCCGATG<br>TGCTCGGCGTAGCCCTTGGC	Used for PCR confirmation of the genotype of the Δ*gra-orf32*Δ*kina-like-pks* mutant
V-pigpks-OF1<br>V-pigpks-IF2<br>V-pigpks-IR3<br>V-pigpks-OR4	TCATCGATGGCCTCCAGCGT<br>TCTTCCACCTGGGGCCGCTC<br>GGTGATGCTGTTGTCGGTGTGTC<br>ACGCGCCCATGACCGTGGAG	Used for PCR confirmation of the genotype of the Δ*gra-orf32*Δ*pig-pks* mutant

Supplementary Table S3. List of the putative genes of 4'-phosphopantetheinyl transferase in the genome of *S. vietnamensis* GIMV4.0001.

Number	Gene ID	Amino acids	Description	Location	Type of predicted pathway	Closest product
1	SVTN_RS01930	246	4'-phosphopantetheinyl transferase	412,960 – 413,697	terpene, NRPS	glycinocin A
2	SVTN_RS03490	223	4'-phosphopantetheinyl transferase	817,499 – 818,170	T1PKS, NRPS, butyrolactone	salinomycin
3	SVTN_RS03505	282	4'-phosphopantetheinyl transferase	819,690 – 820,538	T1PKS, NRPS, butyrolactone	salinomycin
4	SVTN_RS03565	241	4'-phosphopantetheinyl transferase	834,325 – 835,050	T1PKS, NRPS, butyrolactone	salinomycin
5	SVTN_RS18755	218	4'-phosphopantetheinyl transferase	4,175,584 – 4,247,409	T2PKS	granaticin
6	SVTN_RS21115	147	holo-ACP synthase	4,703,192 – 4,703,635	NRPS-like	lankamycin
7	SVTN_RS23020	122	holo-ACP synthase	5,095,059 – 5,095,427	-	-
8	SVTN_RS28640	296	4'-phosphopantetheinyl transferase	6,371,518 – 6,372,408	T2PKS, NRPS, T1PKS	kinamycin
9	SVTN_RS28710	247	4'-phosphopantetheinyl transferase	6,385,933 – 6,386,676	T2PKS, NRPS, T1PKS	kinamycin
10	SVTN_RS28805	220	4'-phosphopantetheinyl transferase	6,414,427 – 6,415,089	T2PKS, NRPS, T1PKS	kinamycin
11	SVTN_RS28845	216	4'-phosphopantetheinyl transferase	6,421,559 – 6,422,209,	T2PKS, NRPS, T1PKS	kinamycin
12	SVTN_RS32290	266	4'-phosphopantetheinyl transferase	7,213,387 – 7,214,187	NRPS	enduracidin

Supplementary Figure 1. Sequence of the codon-optimized *acpS* from the fatty acid biosynthetic pathway of *E. coli* for expression in *Streptomyces*.

ATGgccatcctcgggctcggcaccgacatcgtcgagatcgcgcgcatcgaggcggtgatcgcccgctccggcgacc
gcctcgcccggcgcgtgctgtccgacaacgagtgggcgatctggaagacccaccaccagcccgtgcgcttcctggccaa
gcggttcgccgtcaaggaggccgccgcgaaggccttcggcaccggcatccgcaacggcctggcgttcaaccagttcga
ggtcttcaacgacgagctgggcaagccgcgcctgcggctgtggggcgaggcgctgaagctggccgagaagctcggcg
tggccaacatgcacgtcaccctcgccgacgagcggcactacgcctgcgcgaccgtcatcatcgagagcTGA

Supplementary Figure 2. Sequence of the codon-optimized *sfp* from the surfactin biosynthetic pathway of *Bacillus subtilis* for expression in *Streptomyces*.

ATGaagatctacggcatctacatggaccggccgctgtcccaggaggagaacgagcgcttcatgaccttcatctcccccg
agaagcgggagaagtgccggcgcttctaccacaaggaggacgcccaccgcaccctgctgggcgacgtcctggtgcgc
agcgtgatcagccgccagtaccagctggacaagtcggacatccggttctccacccaggagtacggcaagccctgcatcc
cggacctgcccgacgcgcacttcaacatctcgcactccggccgctgggtcatcggcgccttcgactcgcagcccatcggc
atcgacatcgagaagaccaagccgatctccctggagatcgcgaagcgcttcttcagcaagaccgagtactccgacctcct
cgccaaggacaaggacgagcagaccgactacttctaccacctgtggtcgatgaaggagtccttcatcaagcaggagggc
aagggcctgtcgctcccgctcgacagcttctcggtgcggctgcaccaggacggccaggtgtccatcgagctccccgaca
gccactccccgtgctacatcaagacctacgaggtcgaccccggctacaagatggccgtctgcgccgcgcacccggactt
cccggaggacatcaccatggtctcctacgaggagctgctcTGA

Supplementary Figure 3. Sequence of the codon-Optimized *gra-orf32* for expression in *E. coli*.

ATGcgcaccccacgcggccgcggcccggatccgggccgcgatccgcgtccgggcccggaaccggcgccaagccc
gggcccggaaccaaccccgcgcccgggccgcccactgctggtgcatggcaccgtggaagactgggcgcgtgccgcc
gcgagtcgccgtgcgccgccgcacccgggcgataccgcgcgcctgcgcgcgttacgcgacgaccgtttacgccgtcgc
ttcctggcgagccgcctgttactgcgtaccaccctgggcgcgctggcggatcgcgatccggatggcattgtgctggcgcg
caccgcgctgggccgcccgtatgcgccggatctgccggaactggattttagcctgagtcataccggcagtctgctggcgg
tggccgttgttcgtggtggccgcgtgggcgtggatgcggaacgtccgggccgcccaatgagcgtgctggaacatcgcat
gtgtacgccgcatgaacgcgcgggtctggatgcccgcggtttacgcggcaccgcgcgcgatgcggaactgctgcgcctg
tggaccctgaaagaagcgtatgcgaaagcgttaggcacgggtttacgccacccgccacgcacctttggcctggacccgg
cggacccggcgggccgtccgaccttaccgggcaccctgagtaccgcgacgcgcaccctgccggatggcaccgtggtg
ggcgtggtgagcgcgattggctttgaacgcggcagcagccgcagcgcggatgatgaaccggtgccgTAA

Supplementary Figure 4. Sequence of the codon-optimized *SVTN_RS23020* for expression in *E. coli*.

ATGattattggcgtgggcattgatgtggcggaaattgatcgctttgcggcgagcattgaacgcaccccgggcctgctgca
gcgcctgtttgtggaacgtgaactgttactgccgagcggcgaacgccgcggcccggcgagcttagcggtgcgctttgccg
cgaaagaagcggtggcgaaagcgctgggcgcgccgggcggcctgcattggaccgatgcggaagtgtatgtggaaggc
agcggtcagccacgtctgcgtgtgcgtggcaccgttgccgcccgcgcggcggaactgggcgtgaaacattggcatgtga
gcctgagccatgatgcgggcgtggcgagcgcggtggtgattgcggaaggcTAA

Supplementary Figure 5. Sequence of the codon-optimized *SVTN_RS21115* for expression in *E. coli*.

ATGccggcgcgcccggcggcgccgccggcgggcggcctgggctgggcgaacctgggcatggatattgtgagcgtg
aaccgcgtgcgccgcctgctggcgcagtatggcgaacgctttttgaacgcatgctgaccccgggcgaactggcggattg
ccgcacgagcagcggcctggatgtgctgagcctgtgcggccgcattgcggcgaaagaagcggcgtttaaaaccctgcg
cgtgcgcggccgctttctgccgtggccggatattgtggtgcgccgcagcgaaggcggctggccgctggtggaactgcat
ggcccggcggcggaaatggcggcggaaagcggcattaccgaaattaccgtgagcattagccatgatgtggattatgcgg
tggcggtggcggcgccgattattgcgccgaccccgagcagtagcaacagcTAA

Supplementary Figure 6. the partial biosynthetic cluster of granaticin showing the extended open reading frame of *gra-orf32* in *S. vietnamensis* GIMV4.0001.

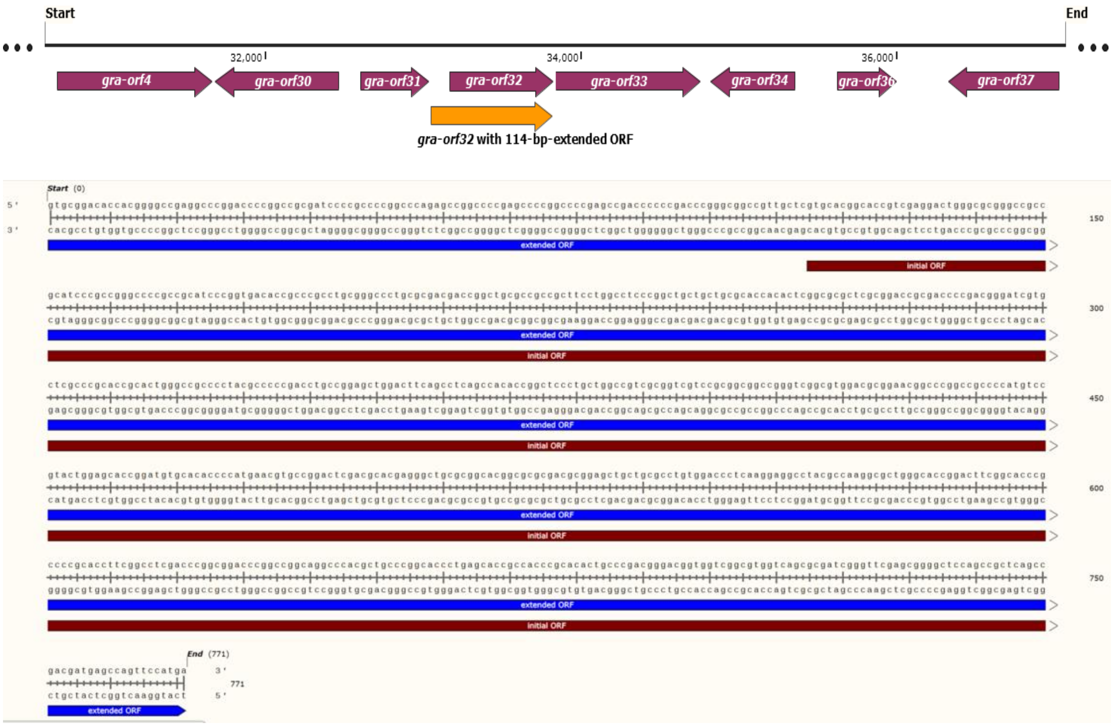

Supplementary Figure 7. Type II PKS clusters associated with Sfp-type PPTase genes. The EFI-EST and EFI-GNT tools (Zallot et al., 2019) were used to do this analysis with Gra-ORF32 used as query protein. E-value and neighbourhood size were set as 5 and l5, respectively. The retrieved clusters were mannually checked. The graphic was edited by Inkscape editor for display.

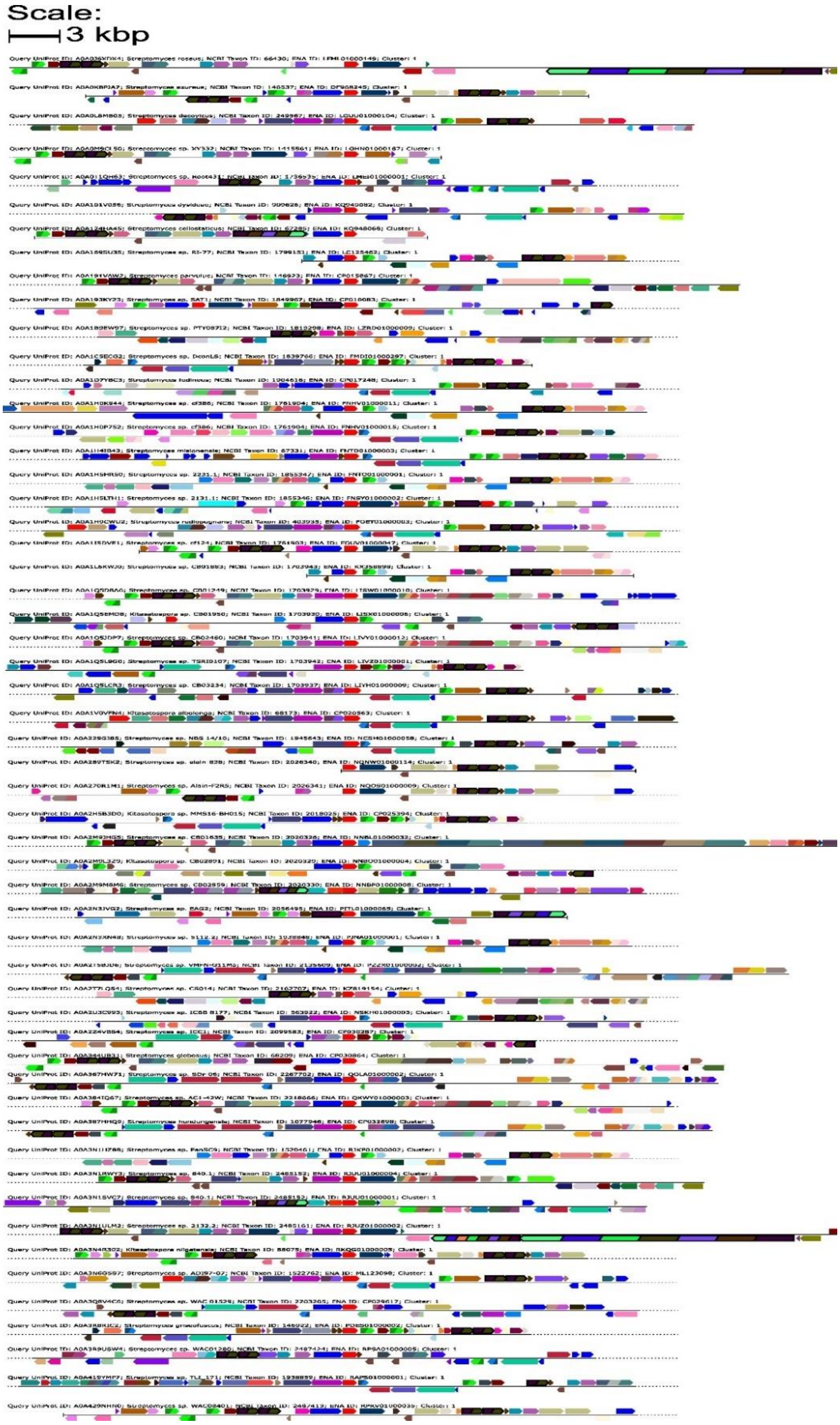

Supplementary Figure 8. Construction of the in-frame deletion mutant strain Δ*gra-orf32* (*SVTN_RS18755*) and confirmation of its genotype by PCR analysis. (**A**) Schematic representation for the deletion of Δ*gra-orf32* (*SVTN_RS18755*) of *Streptomyces vietnamensis* GIMV4.0001 by homologous recombination. Three sets of primers were used to verify the expected in-frame deletion. The fragments amplified with V32LOF/V32RIR, V32LIF/V32ROR or V32LIF/V32RIR from the wild type strain (GIMV4.0001) are 2536, 2387 and 700 bp, respectively. Those from the mutant strain (Δ*gra-orf32*) are 1924, 17557 and 88 bp, respectively. Primers V32LOF and V32ROR stand outside of the homologous arms. (**B**) PCR verification of the in-frame deletion mutant strain Δ*gra-orf32*. w, wild type; m, mutant. Lane 1 and 11, DNA marker DL5000; lane 2, 3 and 4, products amplified with V32LOF/V32RIR; lane 5, 6 and 7, products amplified with V32LIF/V32ROR; lane 8, 9 and 10, products amplified with V32LIF/V32RIR.

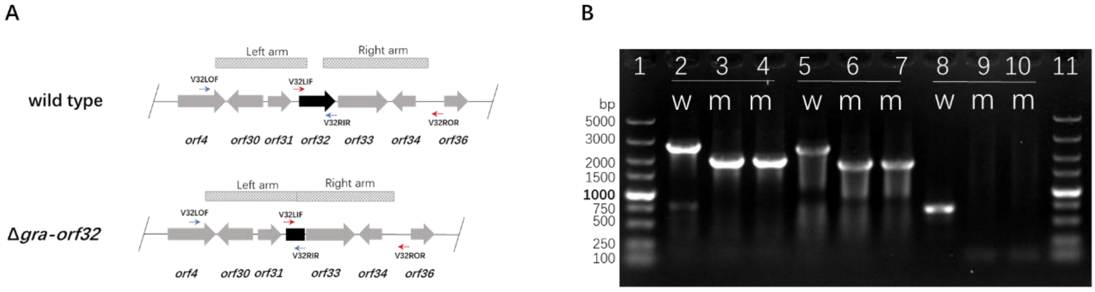

Supplementary Figure 9. Cultures of the deletion and complementation strains of *gra-orf32*.

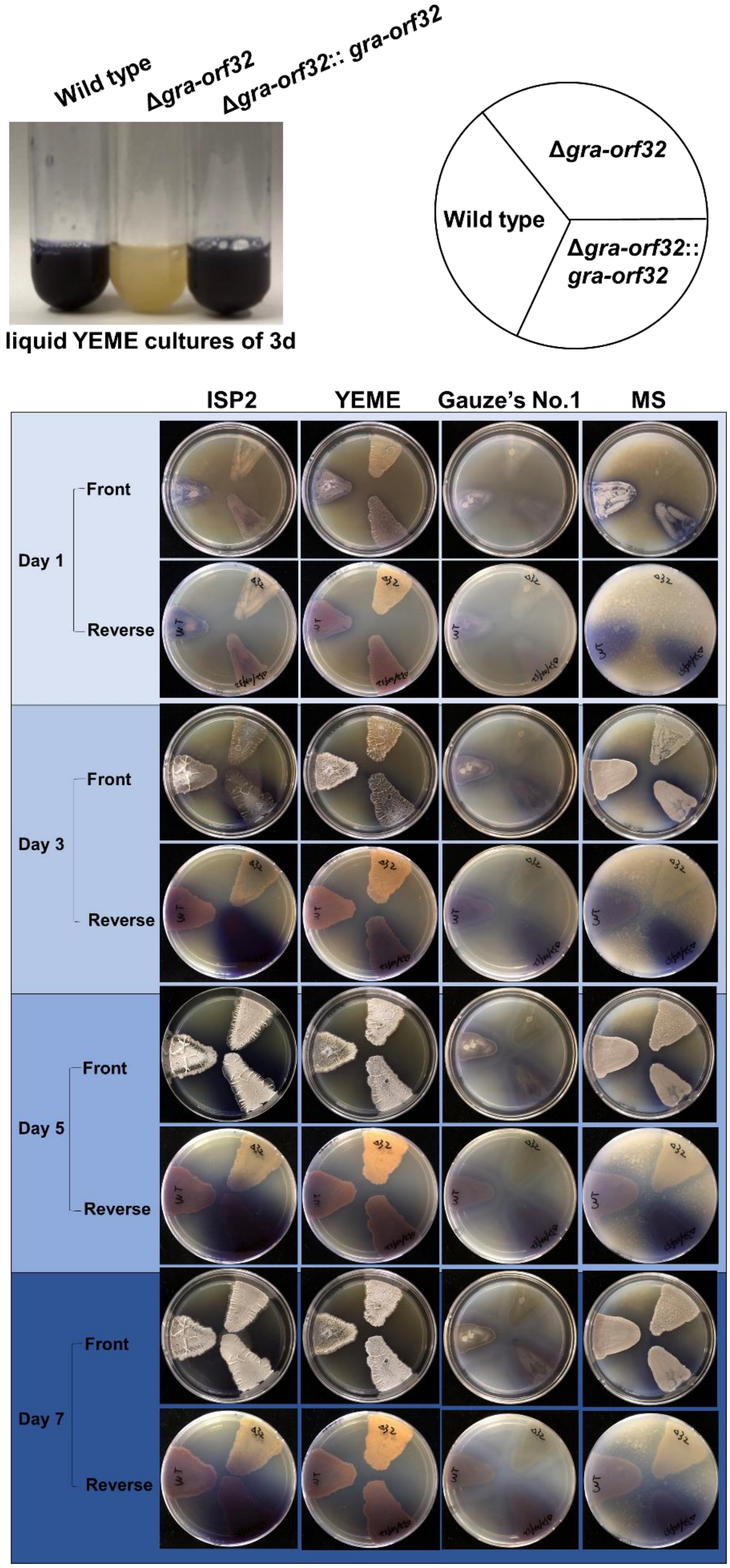

Supplementary Figure 10. PCR verification of complementation of the *gra-orf32* gene and overexpression of other endogenous PPTase genes in the mutant strain Δ*gra-orf32*. Lane 1, DNA marker DL2000; Lane 2, strain with introduction of the blank plasmid pSETAT-KasOp* as a control; Lanes 3 to 5, three separate clones with the desired PPTase genes under the strong promoter KasOp* as indicated on top of each electrophoregram. Vpsetat-F/Vpsetat-F were used as the primer pair.

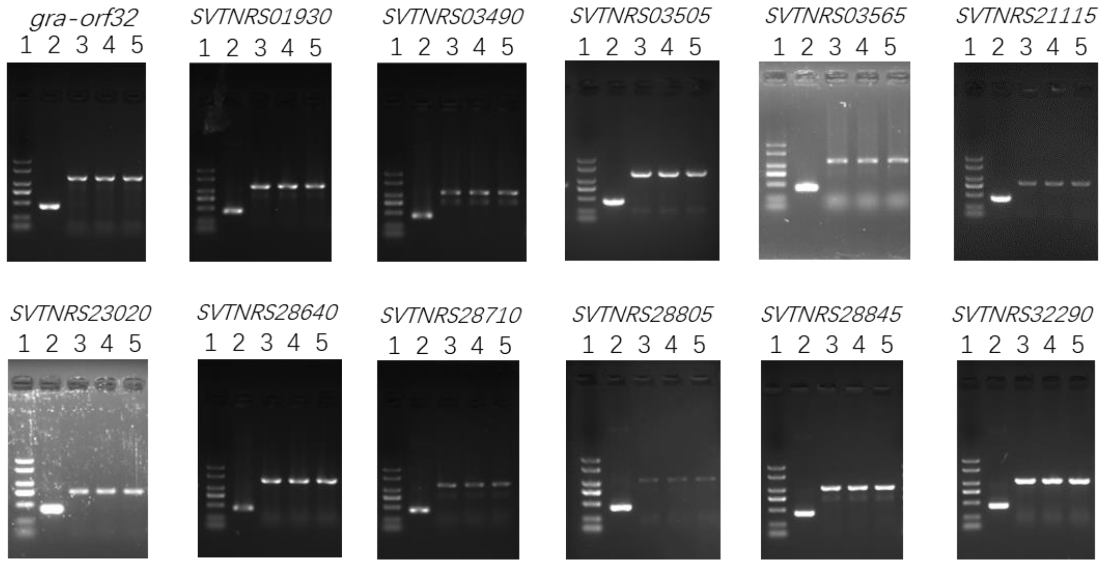

Supplementary Figure 11. The predicted biosynthetic gene clusters of secondary metabolites in the genome of *Streptomyces vietnamensis* GIMV4.0001 by antiSMASH (Blin et al., 2021). Three out of 30 clusters contain type II polyketide synthases. Region 1.11 is the granaticin cluster. The most similar known clusters of regions 1.18 and 1.26 are the clusters of kinamycin and spore pigment with 51% and 83% of genes showing significant similarity, respectively.

dentified secondary metabolite regions using strictness 'relaxed'

IZ_CP010407.1 (Streptomyces vietnamensis)

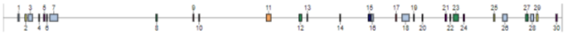

Region	Type	From	To	Most similar known cluster		Similarity
Region 1.1	amglyccycl, butyrolactone	234,459	256,248	pyralomicin 1a	NRP + Polyketide:Modular type I	18%
Region 1.2	lanthipeptide	348,234	370,623	venezuelin	RiPP:Lanthipeptide	100%
Region 1.3	terpene, NRPS	398,540	466,549	glycinocin A	NRP	6%
Region 1.4	ectoine	566,113	576,529	ectoine	Other	100%
Region 1.5	terpene	642,717	663,150	geosmin	Terpene	100%
Region 1.6	butyrolactone	690,816	699,643	coelimycin P1	Polyketide:Modular type I	8%
Region 1.7	T1PKS, NRPS, butyrolactone	740,228	867,785	salinomycin	Polyketide:Modular type I	28%
Region 1.8	CDPS	2,424,610	2,445,359	malacidin A / malacidin B	NRP:Ca+-dependent lipopeptide	5%
Region 1.9	siderophore	3,013,127	3,025,118			
Region 1.10	siderophore	3,104,765	3,115,511	desferrioxamin B	Other	100%
Region 1.11	T2PKS	4,175,584	4,247,409	granaticin	Polyketide:Type II	97%
Region 1.12	NRPS-like	4,695,216	4,736,350	lankamycin	Polyketide	16%
Region 1.13	butyrolactone	4,821,281	4,830,636	scleric acid	NRP	23%
Region 1.14	melanin	5,341,238	5,351,639	istamycin	Saccharide	8%
Region 1.15	other	5,791,545	5,832,648	stambomycin A / stambomycin B / stambomycin C / stambomycin D	Polyketide:Modular type I + Saccharide:Hybrid/tailoring	16%
Region 1.16	LAP, thiopeptide	5,840,626	5,873,649	BD-12	NRP	17%
Region 1.17	siderophore	6,226,796	6,241,184			
Region 1.18	T2PKS, NRPS, T1PKS	6,327,233	6,440,759	kinamycin	Polyketide	51%
Region 1.19	bacteriocin	6,510,573	6,520,614			
Region 1.20	butyrolactone	6,646,523	6,657,473	griseoviridin / fijimycin A	NRP:Cyclic depsipeptide + Polyketide:Trans-AT type I	11%
Region 1.21	terpene	7,009,848	7,035,696	hopene	Terpene	69%
Region 1.22	bacteriocin	7,087,107	7,097,922			
Region 1.23	NRPS	7,140,545	7,222,637	enduracidin	NRP	25%
Region 1.24	terpene	7,298,703	7,318,026	ebelactone	Polyketide	5%
Region 1.25	lanthipeptide	7,780,076	7,799,740			
Region 1.26	T2PKS, lanthipeptide, terpene	7,923,859	7,996,374	spore pigment	Polyketide	83%
Region 1.27	NRPS-like	8,288,248	8,329,035			
Region 1.28	T1PKS, NRPS	8,367,712	8,417,038	heat-stable antifungal factor	NRP + Polyketide	75%
Region 1.29	lanthipeptide	8,457,714	8,484,769	A54145	NRP	5%
Region 1.30	terpene	8,768,489	8,789,237	fortimicin	Saccharide	4%

https://antismash-db.secondarymetabolites.org/output/GCF_000830005.1/index.html#

Region1.18

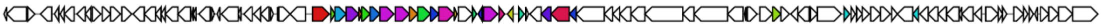

BGC0000236: kinamycin

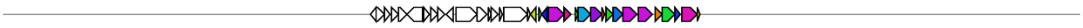

51% of genes show similarity

Region1.26

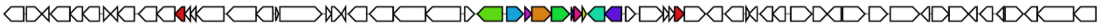

BGC0000271: spore pigment

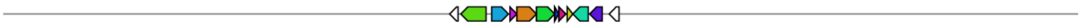

83% of genes show similarity

Supplementary Figure 12. Construction of the double in-frame deletion mutant strain Δ*gra-orf32*Δ*kina-like-pks* and confirmation of its genotype by PCR analysis. (**A**) Schematic representation for the deletion of the minimal PKS genes in the *gra-orf32* deletion background strain of *Streptomyces vietnamensis* (Δ*gra-orf32*) by homologous recombination. Three sets of primers were used to verify the expected in-frame deletion. The fragments amplified with V-kinapks-OF1/V-kinapks-IR3, V-kinapks-IF2/V-kinapks-IR3, V-kinapks-IF2/V-kinapks-OR4 from the *gra-orf32* deletion strain (Δ*gra-orf32*) are 4879, 3077 and 4803 bp, respectively. Those from the double deletion mutant strain (Δ*gra-orf32*Δ*kina-like-pks*) are 2079, 277 and 2003 bp, respectively. Primers V-kinapks-OF1 and V-kinapks-OR4 stand outside of the homologous arms. (**B**) PCR verification of the double deletion mutant strain (Δ*gra-orf32*Δ*kina-like-pks*). m, the double deletion mutant; p, the parent strain Δ*gra-orf32*. Lane 1 and 14, DNA marker DL5000; lane 2, 3, 4 and 5, products amplified with V-kinapks-OF1/V-kinapks-IR3; lane 6, 7, 8 and 9, products amplified with V-kinapks-IF2/V-kinapks-IR3; lane 10, 11, 12 and 13, products amplified with V-kinapks-IF2/V-kinapks-OR4.

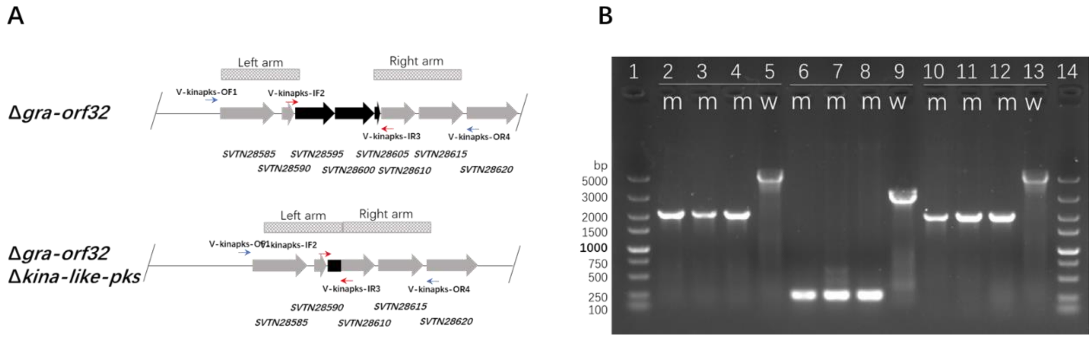

Supplementary Figure 13. Construction of the double in-frame deletion mutant strain Δ*gra-orf32*Δ*pig-pks* and confirmation of its genotype by PCR analysis. (A) Schematic representation for the deletion of the minimal PKS genes of the spore pigment cluster in the *gra-orf32* deletion background strain of *Streptomyces vietnamensis* (Δ*gra-orf32*). Three sets of primers were used to verify the expected in-frame deletion. The fragments amplified with V-pigpks-OF1/V-pigpks-IR3, V-pigpks-IF2/V-pigpks-IR3, V-pigpks-IF2/V-pigpks-OR4 from the *gra-orf32* single deletion strain (Δ*gra-orf32*) are 4728, 2928 and 4719 bp, respectively. Those from the double deletion mutant strain (Δ*gra-orf32*Δ*kina-like-pks*) are 1969, 166 and 1960 bp, respectively. Primers V-pigpks-OF1 and V-pigpks-OR4 stand outside of the homologous arms. (B) PCR verification of the double deletion mutant strain (Δ*gra-orf32*Δ*pig-pks*). m, the double deletion mutant; p, the parent strain Δ*gra-orf32*. Lane 1 and 14, DNA marker DL5000; lane 2, 3, 4 and 5, products amplified with V-pigpks-OF1/V-pigpks-IR3; lane 6, 7, 8 and 9, products amplified with V-pigpks-IF2/V-pigpks-IR3; lane 10, 11, 12 and 13, products amplified with V-pigpks-IF2/V-pigpks-OR4.

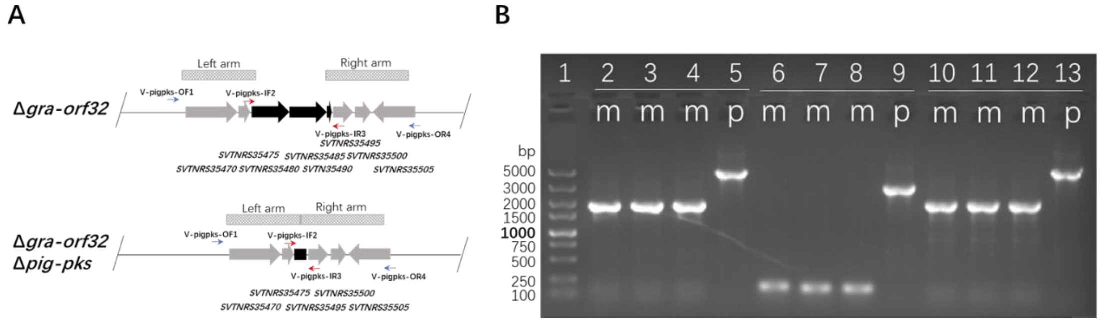

Supplementary Figure 14. Construction of the triple in-frame deletion mutant strain Δ*gra-orf32*Δ*kina-like-pks*Δ*pig-pks* and confirmation of its genotype by PCR analysis. (A) Schematic representation for the deletion of the minimal PKS genes of the spore pigment cluster in the Δ*gra-orf32*Δ*kina-like-pks* double deletion mutant strain. Three sets of primers were used to verify the expected in-frame deletion. The fragments amplified with V-pigpks-OF1/V-pigpks-IR3, V-pigpks-IF2/V-pigpks-IR3, V-pigpks-IF2/V-pigpks-OR4 from the Δ*gra-orf32*Δ*kina-like-pks* mutant are 4728, 2928 and 4719 bp, respectively. Those from the triple deletion mutant strain (Δ*gra-orf32*Δ*kina-like-pks*Δ*pig-pks*) are 1969, 166 and 1960 bp, respectively. Primers V-pigpks-OF1 and V-pigpks-OR4 stand outside of the homologous arms. (B) PCR verification of the triple deletion mutant strain (Δ*gra-orf32*Δ*kina-like-pks*Δ*pig-pks*). m, the double deletion mutant; p, the parent strain Δ*gra-orf32*Δ*kina-like-pks*. Lane 1 and 14, DNA marker DL5000; lane 2, 3, 4 and 5, products amplified with V-pigpks-OF1/V-pigpks-IR3; lane 6, 7, 8 and 9, products amplified with V-pigpks-IF2/V-pigpks-IR3; lane 10, 11, 12 and 13, products amplified with V-pigpks-IF2/V-pigpks-OR4.

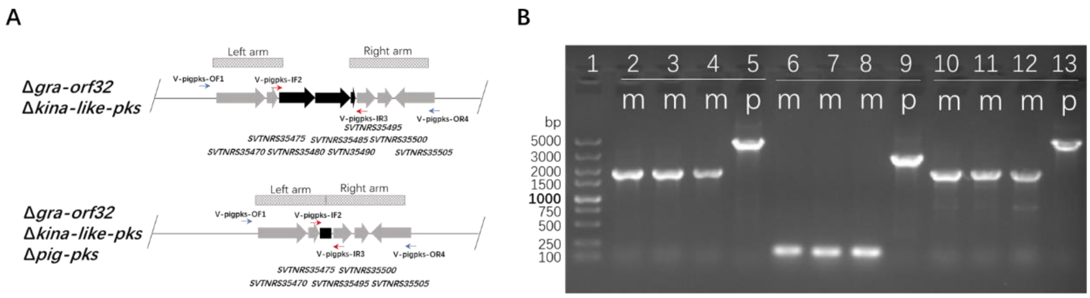

Supplementary Figure 15. PCR verification of introduction of other exogenous PPTase genes into the mutant strain Δ*gra-orf32*. Lane 1, DNA marker DL2000; Lane 2, strain with of the blank plasmid pSETAT-KasOp* as a control; Lanes 3 to 5, three separate clones with the desired PPTase genes under the strong promoter KasOp* as indicated on top of each electrophoregram. Vpsetat-F/Vpsetat-F were used as the primer pair.

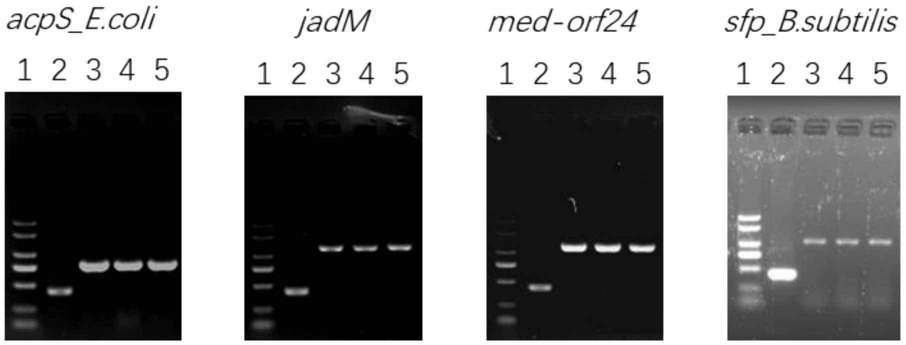

Supplementary Figure 16. Sequence alignment showing the Arg- and Pro-rich N terminuses of the in-cluster Sfp-type PPTases of type II PKSs. Arginine and proline are colored in sky blue. The Arg- and Pro-rich N terminuses are highlighted in red square box.

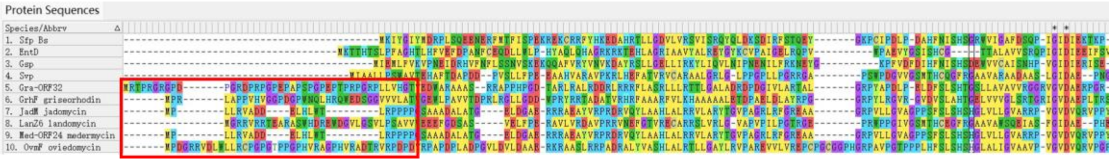

Supplementary Figure 17. Phylogenetic tree of PPTases generated by MEGA 11 (Tamura et al., 2021). PPTases from *S. vietnamensis* and other known PPTase were highlighted in red and pink, respectively.

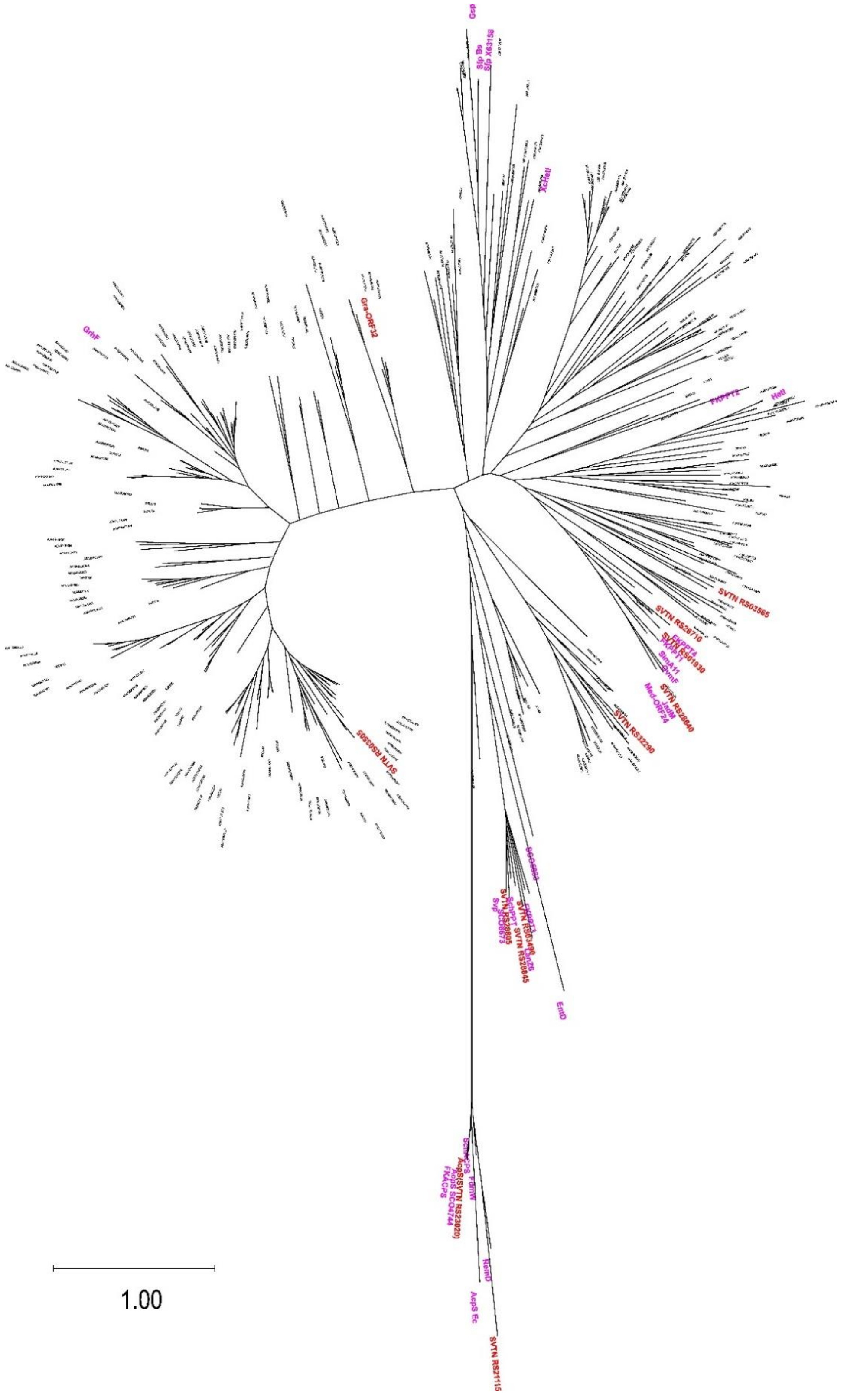

Supplementary Material References

Bierman, M., Logan, R., O'Brien, K., Seno, E.T., Nagaraja Rao, R., and Schoner, B.E. (1992). Plasmid cloning vectors for the conjugal transfer of DNA from *Escherichia coli* to *Streptomyces* spp. *Gene* 116(1)**,** 43-49. doi: 10.1016/0378-1119(92)90627-2.

Blin, K., Shaw, S., Kloosterman, A.M., Charlop-Powers, Z., van Wezel, G.P., Medema, Marnix H., et al. (2021). antiSMASH 6.0: improving cluster detection and comparison capabilities. *Nucleic Acids Research* 49(W1)**,** W29-W35. doi: 10.1093/nar/gkab335.

MacNeil, D.J., Gewain, K.M., Ruby, C.L., Dezeny, G., Gibbons, P.H., and MacNeil, T. (1992). Analysis of *Streptomyces avermitilis* genes required for avermectin biosynthesis utilizing a novel integration vector. *Gene* 111(1)**,** 61-68. doi: 10.1016/0378-1119(92)90603-M

Pan, G., Xu, Z., Guo, Z., Hindra, Ma, M., Yang, D., et al. (2017). Discovery of the leinamycin family of natural products by mining actinobacterial genomes. *Proceedings of the National Academy of Sciences* 114(52)**,** E11131-E11140. doi: 10.1073/pnas.1716245115.

Sun, Y., Hong, H., Samborskyy, M., Mironenko, T., Leadlay, P.F., and Haydock, S.F. (2006). Organization of the biosynthetic gene cluster in Streptomyces sp. DSM 4137 for the novel neuroprotectant polyketide meridamycin. *Microbiology* 152(12)**,** 3507-3515. doi: https://doi.org/10.1099/mic.0.29176-0.

Takiff, H.E., Baker, T., Copeland, T., Chen, S., and Court, D. (1992). Locating essential *Escherichia coli* genes by using mini-Tn10 transposons: the *pdxJ* operon. *Journal of bacteriology* 174(5)**,** 1544-1553. doi: https://doi.org/10.1128/jb.174.5.1544-1553.1992.

Tamura, K., Stecher, G., and Kumar, S. (2021). MEGA11: molecular evolutionary genetics analysis version 11. *Molecular Biology and Evolution* 38(7)**,** 3022-3027. doi: 10.1093/molbev/msab120.

Zallot, R., Oberg, N., and Gerlt, J.A. (2019). The EFI web resource for genomic enzymology tools: leveraging protein, genome, and metagenome databases to discover novel enzymes and metabolic pathways. *Biochemistry* 58(41)**,** 4169-4182. doi: 10.1021/acs.biochem.9b00735.

Zhu, H.-h., Guo, J., Yao, Q., Yang, S.-z., Deng, M.-r., Phuong, L.T.B., et al. (2007). *Streptomyces vietnamensis* sp. nov., a streptomycete with violet blue diffusible pigment isolated from soil in Vietnam. *International Journal of Systematic and Evolutionary Microbiology* 57(8)**,** 1770-1774. doi: 10.1099/ijs.0.64774-0.
